# Linggui Zhugan Decoction activates the SIRT1-AMPK-PGC1α signaling pathway to improve mitochondrial and oxidative damage in rats with chronic heart failure caused by myocardial infarction

**DOI:** 10.3389/fphar.2023.1074837

**Published:** 2023-04-05

**Authors:** Siyi Yu, Hang Qian, Dawei Tian, Mingming Yang, Dongfeng Li, Hao Xu, Jishun Chen, Jingning Yang, Xincai Hao, Zhixin Liu, Jixin Zhong, Handong Yang, Xinlong Chen, Xinwen Min, Jun Chen

**Affiliations:** ^1^ Sinopharm Dongfeng General Hospital (Hubei Clinical Research Center of Hypertension), Hubei University of Medicine, Shiyan, Hubei, China; ^2^ Jiujiang No. 1 People’s Hospital, Affiliated Jiujiang Hospital of Nanchang University, Jiujiang, Jiangxi, China; ^3^ Department of Immunology, School of Basic Medicine, Hubei University of Medicine, Shiyan, Hubei, China; ^4^ Hubei Key Laboratory of Wudang Local Chinese Medicine Research (Hubei University of Medicine), Shiyan, Hubei, China; ^5^ Institute of Virology, Hubei University of Medicine, Shiyan, Hubei, China; ^6^ Department of Rheumatology and Immunology, Tongji Hospital, Huazhong University of Science and Technology, Wuhan, Hubei, China; ^7^ Yunxi Hospital of Chinese Medicine, Shiyan, Hubei, China

**Keywords:** mitochondria, heart failure, myocardial infarction, animal experiments, traditional Chinese medicine

## Abstract

**Objective:** To investigate the effects of Linggui Zhugan Decoction on mitochondrial and oxidative damage in rats with chronic heart failure after myocardial infarction and the related mechanisms.

**Methods:** Chronic heart failure after myocardial infarction was established by coronary artery ligation. Heart failure rats were randomly divided into three groups: Model group (*n* = 11), Linggui Zhugan Decoction group (*n* = 12), and captopril group (*n* = 11). Rats whose coronary arteries were only threaded and not ligated were sham group (*n* = 11). Cardiac function, superoxide dismutase (SOD), malondialdehyde (MDA) contents, soluble growth-stimulating expression factor (ST2), and N-terminal B-type brain natriuretic peptide precursor (NTproBNP) levels were analyzed after treatment. Moreover, the level of mitochondrial membrane potential was detected by JC-1 staining, the ultrastructural of myocardial mitochondria were observed by transmission electron microscopy. The related signal pathway of silent information regulator factor 2-related enzyme 1 (SIRT1), adenylate activated protein kinase (AMPK), phosphorylated adenylate activated protein kinase (p-AMPK), and peroxisome proliferator-activated receptor *γ* coactivator 1α (PGC-1α) is an important pathway to regulate mitochondrial energy metabolism, and to initiate mitochondrial biogenesis. The expression level was detected by Western blot and reverse transcription to explore the mechanism of the decoction.

**Results:** Compared with the model rats, Linggui Zhugan Decoction significantly improved cardiac function (*p* < 0.05), reduced MDA production (*p* < 0.01), increased SOD activity (*p* < 0.05), reduced ST-2(*p* < 0.01), and NT-proBNP(*p* < 0.05) levels, increased mitochondrial membrane potential, and improved mitochondria function. In addition, Linggui Zhugan Decoction upregulated the expression of SIRT1, p-AMPK, PGC-1α protein, and mRNA in cardiac myocytes.

**Conclusion:** Linggui Zhugan Decoction can improve the cardiac function of heart failure rats by enhancing myocardial antioxidant capacity and protecting the mitochondrial function, the mechanism is related to activating SIRT1/AMPK/PGC-1α signaling pathway.

## 1 Introduction

Chronic heart failure (CHF) is a complex clinical syndrome and an end-stage cardiovascular disease. Although great progress has been achieved in understating the pathophysiology of the disease, the global incidence and mortality rates of CHF remain high. The main reason for this is an increase in the aging population. HF still represents a severe public health burden and has a huge impact on healthcare costs (Savarese and Lund, 2017). According to statistics, it has been estimated that the prevalence of heart failure in the United States will increase by 25% by 2030, while the treatment expenditure for heart failure will increase by two times (Tayal et al., 2017). Thus, searching for new drugs is crucial for improving prognosis and improving long-term survival in patients with heart failure.

CHF formation is regulated by a complex pathophysiological process (Braunwald, 2013). The energy metabolism disorder is one of the most important reasons for CHF (Lopaschuk et al., 2021). The existing research results (Bertero and Maack, 2018) have shown that myocardial energy metabolism disorders can induce heart failure, which further aggravates energy metabolism disorders. It has been suggested that mitochondria dysfunction has an important role in the occurrence and development of heart failure. Silent mating type information regulation 2 homolog 1 (SIRT1) is a histone deacetylase dependent on nicotinamide adenine dinucleotide, which can deacetylate many proteins and has a vital role in oxidative stress (Lee et al., 2018). Peroxisomeproliferator-activated receptor*?* coactivator-1α (PGC-1α) is the deacetylation substrate of SIRT1, and the stimulation of SIRT1 can promote the activation of PGC-1α and, in turn, inhibit oxidative stress damage (Gurd, 2011). Moreover, adenosine activated protein kinase (AMPK), known as “cell energy receptor,” is the key molecule of biological energy metabolism regulation; it can improve mitochondrial dysfunction through PGC-1α and enhance AMPK activity, promoting cell survival (Herzig and Shaw, 2018). Thus, it is believed that SIRT1/AMPK/PGC-1α signaling pathway has a major role in the defense against oxidative stress and mitochondrial dysfunction.

Over recent years, research on traditional Chinese medicine has been rapidly increasing. Chinese medicine has the characteristics of multi-site synergy, offering anti-oxidative stress, anti-inflammation properties, improving ventricular remodeling, reducing myocardial fibrosis, and promoting angiogenesis (Xu et al., 2005; Wang et al., 2013; Cao et al., 2015; Zheng et al., 2019; Bu et al., 2020). Linggui Zhugan Decoction, derived from Synopsis of Golden Chamber by Zhang Zhongjing and composed of Fuling [the dry fungus nucleus of *Poria cocos* (Schw.) Wolf], Guizhi [the dry twigs of Neolitsea cassia (L.) Kosterm. (Lauraceae)], Baizhu [the dry rhizome of Atractylodes macrocephala Koidz. (Asteraceae)], and Gancao [the dry root and rhizome of Glycyrrhiza glabra L. (Fabaceae)], is a classical Chinese prescription often used as a complementary treatment to conventional western medicine for heart failure (Fu et al., 2010). The common research on Linggui Zhugan Decoction is limited to reducing myocardial oxygen consumption, such as slowing down heart rate and lowering blood pressure. Whether this decoction can treat heart failure by increasing oxygen supply has not been studied. In this study, we examined the effect and protective mechanism of Linggui Zhugan Decoction on cardiac function in CHF rats through mitochondria, which could provide a new theoretical basis and therapeutic target for the prevention and treatment of heart failure with Linggui Zhugan Decoction.

## 2 Materials and methods

### 2.1 Animals

Sixty SPF male SD rats, aged 6 weeks, weighing 210 ± 10 g, were provided by Beijing Weitong Lihua Experimental Animal Technology Co., Ltd (experimental animal certificate No. SCXK (Beijing) 2016-0006). All the animals were housed in a specific-pathogen-free (SPF) environment with a temperature of 25°C ± 2°C, relative humidity of 55%-70%, and a light/dark cycle of 12/12 h. All animal studies (including the rats euthanasia procedure) were done in compliance with the regulations and guidelines of Hubei University of Medicine institutional animal care and conducted according to the AAALAC and the IACUC guidelines (approval number: Dong (Fu) No.2020-Shi 035 of Hubei University of Medicine).

### 2.2 Medicines

Fuling (batch number: 200901), Guizhi (batch number: 200701), Baizhu (batch number: 201101), and Gancao (batch number: 201101), according to the ratio of 4:3:3:2, purchased from the outpatient pharmacy of Dongfeng General Hospital of Traditional Chinese Medicine, identified by Hubei Qiangkang Herbal Pieces Co., Ltd.; strain identification conforms to the standard of traditional Chinese medicine. Add 10 times the volume of distilled water to soak the Chinese herbal pieces for 30 min. Then boil 3 times, mix the obtained 3 times filtrate, filter through gauze, rotate and concentrate to prepare a liquid containing 1 g/mL crude drug, which was stored in a refrigerator at 4°C. Captopril (purchased at TargetMol, lot number T1462). In order to ensure the quality and stability of the decoction, the four chemical constituents in the prepared Linggui Zhugan Decoction, including Pachymic acid (from Fuling), Cinnamic acid (from Guizhi), Atractylenolide III (from Baizhu) and Glycyrrhizic acid (from Gancao), were determined by HPLC. In addition, we also preliminarily measured the blood concentrations of these four chemical components in rats after Linggui Zhugan Decoction intervention for 1 h.

### 2.3 Reagents and instruments

Cinnamic acid (140-10-3), Glycyrrhizic acid (1,405-86-3), Atractylenolide III (73030-71-4), Pachymic acid (29070-92 -6), all purchased from Beijing Zhongke Quality Inspection Biological Co., Ltd. MDA and SOD kits were purchased from Nanjing Jiancheng Bioengineering Institute, with batch number A003-2 and A001-3, respectively; NT-proBNP kit was acquired from Immunoway (product number KE1761), and ST2 kit was purchased from Abcam (product number ab255716). p-AMPK antibody was obtained from Cell Signaling, (Batch No. 2,535); AMPK antibody was bought from Immunoway (Batch No. YT0216); SIRT1 antibody was purchased from Santa Cruz (Batch No. sc-74465); PGC-1α antibody was obtained from Abcam company (product number ab191838); GAPDH antibody was purchased from antGene (product number ANT325); Isoflurane was purchased from RWD Biotechnology Co., Ltd. (Batch No. R510-22); Pentobarbital sodium was purchased from MERCK(Product No. Y0002194).

EClassical 3,100 high performance liquid chromatograph (Elite, China); SupersilODS25um high performance liquid chromatography column (Elite, China); Small animal anesthesia machine (RWD, China); Vevo2100 ultra-high-resolution small animal color Doppler ultrasound real-time imaging system (Visual Sonics, Canada); MQX-200 microplate reader (Bio TEK INC, United States); Transmission electron microscope (Hitachi, Japan); U-0080D ultraviolet spectrophotometer (Hitachi, Japan); UPV INC gel imaging system (Gene company); MF43 microscope (Mingmei Radio and Television Technology, Guangzhou) were also used in this study.

### 2.4 Model preparation

After 7 days of adaptive feeding, 60 rats were randomly divided into sham operation group (13 rats) and model group (47 rats). Chronic heart failure after myocardial infarction was established by ligation of the left coronary artery. Briefly, rats were fasted for 12 h before the operation and were only allowed to drink water. Then 40 mg/kg pentobarbital sodium intraperitoneal injection anesthesia was applied, and rats were placed in a supine position and given fixed tracheal intubation, mechanical ventilation (respiratory rate 70-90 times/min, respiratory ratio 1:2). After precardiac skin disinfection, the left third to fourth intercostal space thoracotomy was performed to expose the heart. The left coronary artery was ligated with a 5–0 suture needle at 1–1.5 mm below the left atrial appendage and pulmonary cone (the sham operation group was only sutured without ligation), and after the thoracic cavity was closed. Next, the chest wall was hierarchically sutured, and the ventilator was closed. Consequently, rats were placed on the insulation blanket and then returned to the cage after restoring spontaneous breathing and waking up.

Each rat received an intraperitoneal injection of 400,000 U penicillin for three consecutive days to prevent infection. The ischemia and whitening of myocardial tissue at the lower end of the ligation line were immediately observed after ligation, and the ST segment of lead II ECG was significantly elevated as a sign of successful ligation (there was no above performance in the sham operation group after the ligation). After 1 week of routine feeding, the feed was halved, and the rats were forced to swim once a day until the chronic heart failure model was successfully established.

### 2.5 Grouping and processing

At the fourth week after the operation, if the rats gradually showed decreased activity, loss of appetite, loss of weight, accelerated respiratory heartbeat, cyanosis at the bottom of the paw, and left ventricular ejection fraction (LVEF %) < 45% by ultrasonic cardiogram, the heart failure model was considered to be successfully established (Zhang et al., 2013). During the modeling process, 10 rats died (including 2 rats with sham operation), 5 rats were excluded, and 34 rats with successful heart failure were randomly divided into three groups: Model group (*n* = 11), Linggui Zhugan Decoction group (*n* = 12) and captopril group (*n* = 11). Linggui Zhugan Decoction group and captopril group were treated with Linggui Zhugan Decoction group (5.4 g/(kg d) (crude herbs))and captopril group (6.75 mg/(kg d)), respectively, according to the clinical daily dose of 70 kg body weight, and converted dose based on human and animal body surface area. We also refer to the doses of other related experiments. Chen et al. (2022) designed experiments, using a low dose of 4.29 g/kg and a high dose of 8.58 g/kg of Lingguizhugan decoction to treat heart failure mice prepared by aortic coarctation; Wang et al. used 6.6 g/kg (crude herbs) LGSGT to treat adriamycin-induced heart failure mouse model (Wang et al., 2020). The sham operation group and the model group were given intragastric administration of an equal amount of distilled water. Rats in each group were given intragastric administration at the fifth week after the operation, once a day, for a total of 6 weeks. During the experiment, one rat in the sham operation group, two rats in the model group, two in the Chinese medicine group, and one in the western medicine group died. Captopril is an angiotensin converting enzyme inhibitor (ACEI), which is currently widely used as a guideline-recommended first-line drug for the treatment of heart failure (McDonagh et al., 2021). Previous studies have shown that ACEI can help maintain cardiac energy balance (Schultheiss et al., 1990). Captopril has also been shown to improve myocardial energy metabolism by maintaining mitochondrial function, restoring mitochondrial oxygen consumption and reducing cardiac preload (Sanbe et al., 1995; Kojic et al., 2011). Therefore, captopril-intervened heart failure rats were set as the positive control in this experiment.

### 2.6 Detection indicator

#### 2.6.1 HPLC

Using acetonitrile (A)-0.2% phosphoric acid aqueous solution (B) as the mobile phase gradient elution, the elution gradient is as follows: 0–10 min, 30%–50%A; 10–20 min, 50%–70%A; 20–25 min, 70%–82%A; 25–50 min, keep 82%A. The flow rate is 1.0 mL/min, the detection wavelength is 237 nm, the column temperature is 30°C.

#### 2.6.2 General state observation

The body weight of the rats was measured, and the mental state, activity, color of skin, skin gloss, loss of hair, and eating were evaluated.

#### 2.6.3 Echocardiography

Rates were first anesthetized with isoflurane inhalation (induction 4% 2 L/min, maintenance 2% 1.5 L/min), keeping the heart rate of rats at 300–400 times per minute, then fixed on table. M-mode echocardiography was then obtained by placing the M-type sampling line perpendicular to the ventricular septum and left posterior ventricular wall at the level of rat papillary muscle (at the maximum left ventricular diameter). The left ventricular ejection fraction (LVEF%), the left ventricular fraction shortening (LVFS%), the left ventricular end-diastolic diameter (LVIDd), and the left ventricular internal dimension systole (LVIDs) were continuously measured for five cardiac cycles, after which the average values were calculated. The relevant calculation formula is as follows:

$$LV\;vol\left(d,s\right)=\left(\frac{7.0}{2.4+LVID;\left(d,s\right)}\right)\times LVID;{\left(d,s\right)}^{3}$$

$$LVEF\%=100\times \left(\frac{LV\;vol;d-LV\;vod,s}{LV\;vol,d}\right)$$

$$LVFS\%=100\times \left(\frac{LVID;d-LVID,s}{LVID,d}\right)$$

#### 2.6.4 Exhaustive swimming time

Before anesthesia, 10% of the lead weight of the rat was tied to the tail of the rat. Then, the rats were placed in a water tank with a water depth of 50 cm and a water temperature of 30°C for an exhaustive swimming experiment. The standard of exhaustive swimming was that the head of the rats did not float to the water surface for 20 s after submerging, and the exhaustive time of the rats was recorded.

#### 2.6.5 Serum levels of NT-proBNP, ST-2, SOD, and MDA detection

After the rats were sacrificed by intravenous injection of 150 mg/kg pentobarbital sodium, take 5 mL of rat abdominal aorta blood and centrifugate at 4°C 3000 r/min for 15 min, and the serum was stored at-80°C. The contents of MDA, SOD, NT-proBNP, and ST-2 in serum were detected according to the kit instructions. NT-proBNP and ST-2 were detected by ELISA, SOD by WST-1, MDA by thiobarbituric acid (TBA).

#### 2.6.6 Holistic quality index (HW/BW)

The heart was quickly removed. Hearts were then placed in 10% KCL, washed with PBS, after which the aorta was cleared, and the heart weight (HW) and the mass heart index were calculated by heart weight (HW)/body weight (BW).

#### 2.6.7 Mitochondria JC-1 staining

After the heart was collected, 1 mm^3^ of the apical myocardium was cut. Then, the mitochondria were extracted according to the instructions of the mitochondrial kit, and the mito solution was used to resuspend the mitochondrial precipitation (Yang et al., 2018; Wei et al., 2020). The operation was performed following the mitochondrial membrane potential test kit (JC-1). When the mitochondrial membrane potential is high, JC-1 can be seen in the mitochondrial matrix and form a polymer to produce red fluorescence; if the mitochondrial membrane potential is low, this is not observed. JC-1 was a monomer at this time to produce green fluorescence. The relative proportion of red and green fluorescence was used to measure the proportion of mitochondrial depolarization.

#### 2.6.8 Pathological section making and staining

After abdominal aorta blood collection, the chest was opened, the pericardial was cut, and the appendages were removed. The heart was repeatedly washed with ice saline solution until the solution became clear and bright, and the water was sucked into 4% neutral formaldehyde solution by filter paper. After ethanol dehydration and paraffin embedding, hearts were placed in a continuous slice machine. After dewaxing and treating with xylene, HE staining and Masson staining was performed. Finally, a light microscope was used for observation. Myocardial tissue collagen volume fraction (CVF = collagen area/total area) was performed on Masson-stained images using Image J image analysis software.

#### 2.6.9 Electron microscope

The heart tissue of rats was cut into about 1–2 mm^3^ pellets and fixed with 2.5% glutaraldehyde for more than 2 h. After dehydration and embedding, the samples were placed overnight in a 37°C oven, then placed in a 45°C oven for 12 h, and finally placed in a 60°C oven for 48 h. The tissue slicer was used to cut the 60 nm ultra-thin section, and the samples were stained with acetate-lead citrate. The samples were photographed by transmission electron microscope.

#### 2.6.10 Western blot

The myocardial tissue proteins of rats were taken and lysed with protein lysate. The total proteins were extracted. Then, electrophoresis, membrane transfer, and blocking were successively performed. Samples were then incubated with primary antibody at 4°C overnight and secondary antibody for 1 h to detect the protein expression levels of Sirt1, AMPK, p-AMPK, PGC-1α, NRF1, and TFAM, in the myocardium. After ECL coloration, the images were analyzed by ImageJ software.

#### 2.6.11 Reverse transcription-polymerase chain reaction (RT-PCR)

The collected myocardial tissue was ground with Trizol solution. The total RNA of myocardial tissue was isolated and extracted in accordance with the operating instructions of the Trizol reagent. After synthesizing the first-strand cDNA, the levels of Sirt1, PGC-1α, NRF1, and TFAM were detected by RT-PCR. The primer sequence was synthesized by the Shanghai Shenggong Biological Company. The specific sequence is as follows (Table 1). The reaction conditions were: Pre-denaturation: 94°C 5 min; pCR reaction: 94°C 30 s, 58°C 30 s, 72°C 30 s; annealing at 72°C for 3 min and 40 cycles. Then, the amplification curve and the melting curve were confirmed, and the expression levels of each gene were evaluated by 2^−△△CT^ method.

**TABLE 1:** Primer sequences used for the RT-PCR.

Gene symbol	Primer sequence (5ʹ–3ʹ)
PGC-1α	Forward:CGGTGGATGAAGACGGATTGCC
	Reverse:ATTGTAGCTGAGCTGAGTGTTGGC
Sirt1	Forward:GCTCGCCTTGCTGTGGACTTC
	Reverse:GTGACACAGAGATGGCTGGAACTG
NRF1	Forward:AGATGCTAATGGCCCAGATG
	Reverse:AGCTCTGCCTGGTTGTTTGT
TFAM	Forward:GCTCGCCTTGCTGTGGACTTC
	Reverse:GTGACACAGAGATGGCTGGAACTG
β-actin	Forward:TGTCACCAACTGGGACGATA
	Reverse:GGGGTGTTGAAGGTCTCAAA

### 2.7 Statistical analysis

SPSS 22.0 software was used to analyze the data. The measurement data were expressed as mean ± standard deviation. One-way ANOVA was used to compare the normal distribution data between groups, and the LSD method was used to compare the data between groups. Non-normal distribution data were used to compare the differences between groups by a non-parametric test. A *p* < 0.05 indicated that the difference was statistically significant.

## 3 Results

### 3.1 Contents of four active ingredients in Linggui Zhugan Decoction

**FIGURE 1:**
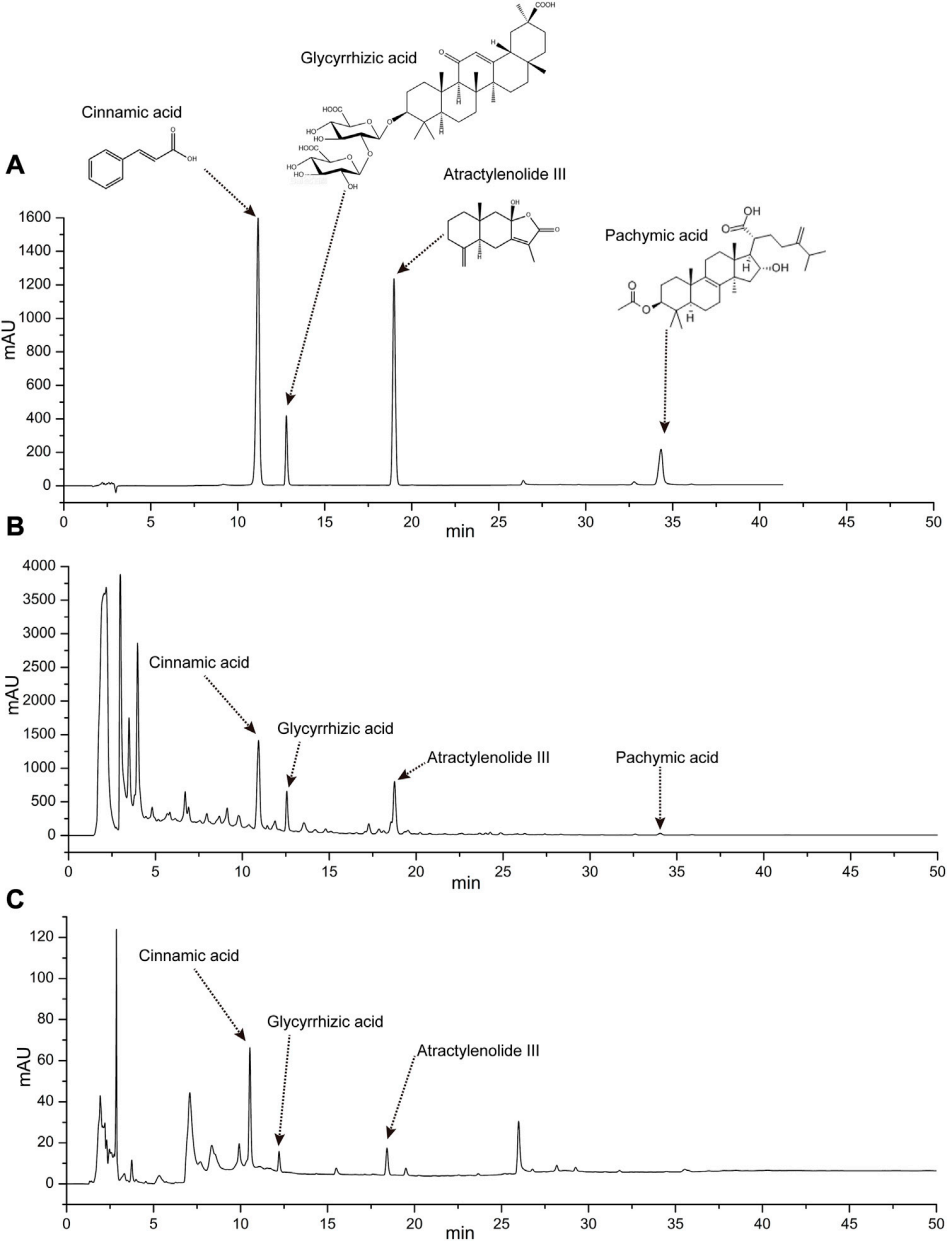
Determination of four chemical constituents of cinnamic acid, glycyrrhizic acid, atractylenolide III, and pachymic acid by HPLC. **(A)** High performance liquid chromatogram of the four chemical composition standards. **(B)** High performance liquid chromatogram of Linggui Zhugan Decoction. **(C)** High performance liquid chromatogram of rat serum after 1 h gavage of Linggui Zhugan decoction.

According to the peak area, it was calculated that the Linggui Zhugan Decoction contains 18.68 ug/mL pachymic acid, 153.49 ug/mL cinnamic acid, 67.47 ug/mL atractylenolide III and 621.34 ug/mL glycyrrhizic acid. It can be seen that glycyrrhizic acid is the most abundant component in Linggui Zhugan Decoction. The four components in the rat serum were measured 1 h after administration, the serum contained 12.55 ug/mL cinnamic acid, 2.90 ug/mL atractylenolide III, and 27.07 ug/mL glycyrrhizic acid. Pachymic acid may be difficult to detect due to insufficient HPLC sensitivity or low concentrations (Figure 1).

### 3.2 Linggui Zhugan Decoction inhibits myocardial remodeling and improves cardiac function in rats with heart failure caused by myocardial infarction

**FIGURE 2:**
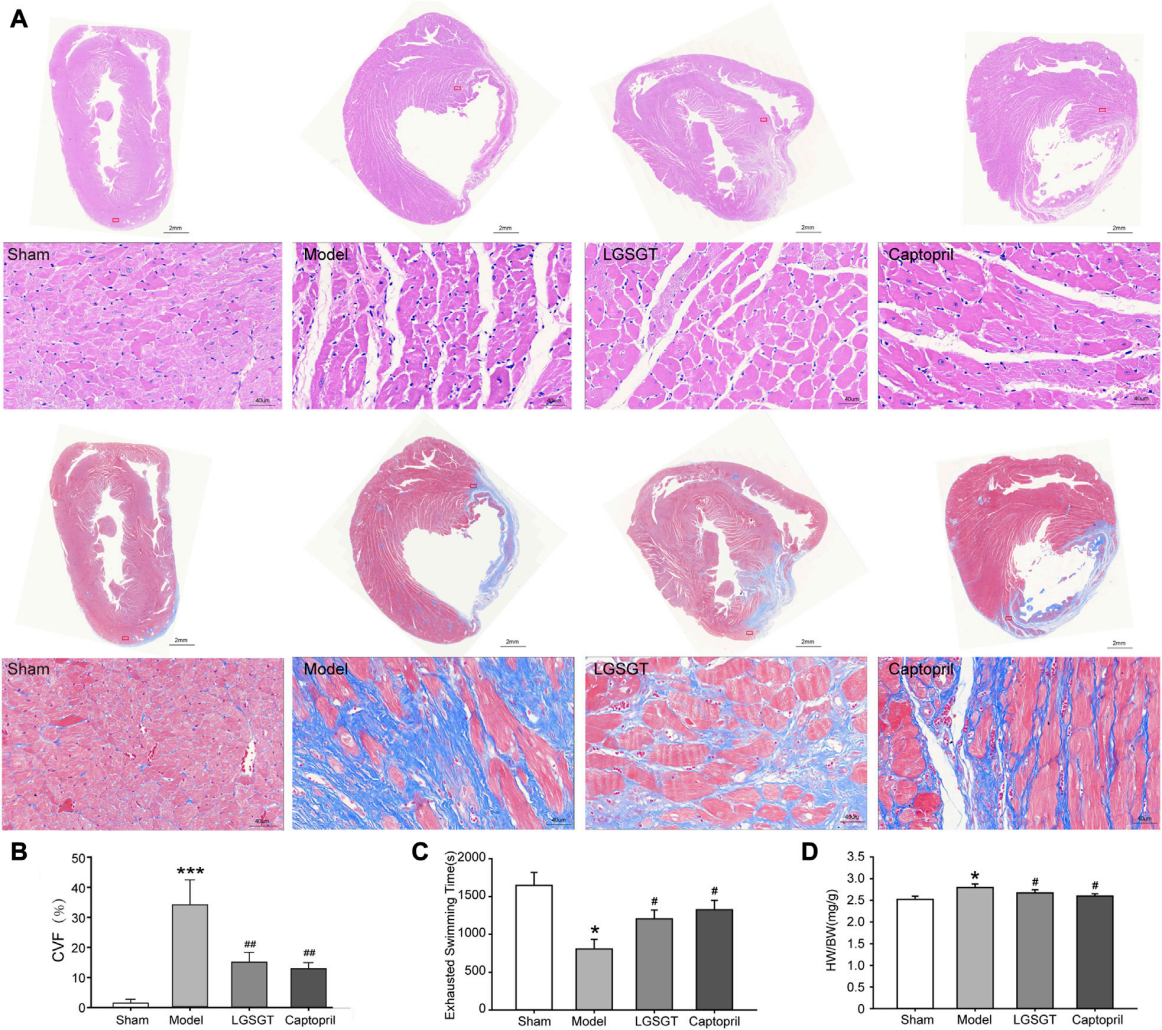
Linggui Zhugan Decoction protects the myocardium from myocardial injury and increases exercise tolerance in rats with chronic heart failure after myocardial infarction. **(A)** HE staining and Masson staining of myocardial tissue in each group. **(B)** The collagen volume fraction in each group (*n* = 6). **(C)** Exhaustive swimming method to evaluate exercise tolerance in rats with heart failure (*n* = 8). **(D)** The heart weight index in each group (heart weight/body weight). Asterisks indicate significant difference as compared with the sham group (*n* = 8). *p < 0.05 vs. sham group; ^#^ *p* < 0.05 vs. model group.

As shown in Figure 2A **,** HE staining showed that the myocardial cells in the sham operation group with intact myocardial fibers and no degeneration or necrosis were structurally regular and neatly arranged. The cardiomyocytes in the model group had different morphological sizes and loose cell arrangements. Some myocardial fibers showed disorganization, and necrosis with infiltration of inflammatory cells was seen in myocardial fibers and interstitium. The degree of myocardial structural damage in the Linggui Zhugan Decoction group and captopril group was significantly improved compared to the model group, and the myocardial fibers were neatly arranged. Masson staining showed that the sham operation group had almost no or only a small amount of collagen fibrous tissue and was evenly distributed. In the model group, a large number of flaky fibrous tissues, severe myocardial fibrosis. Compared with the model group, the collagen fibers in the infarct area and the infarct edge area in the Lingguizhugan decoction group and the captopril group were significantly reduced, and the myocardial cells were arranged more closely. As shown in Figure 2B, quantitative comparison using collagen volume fraction showed that the CVF of the model group was significantly increased compared with that of the sham-operated group (*p* < 0.001). Compared with the model group, the degree of myocardial fibrosis in the captopril group and Linggui Zhugan decoction group was significantly reduced (*p* < 0.01).

**FIGURE 3:**
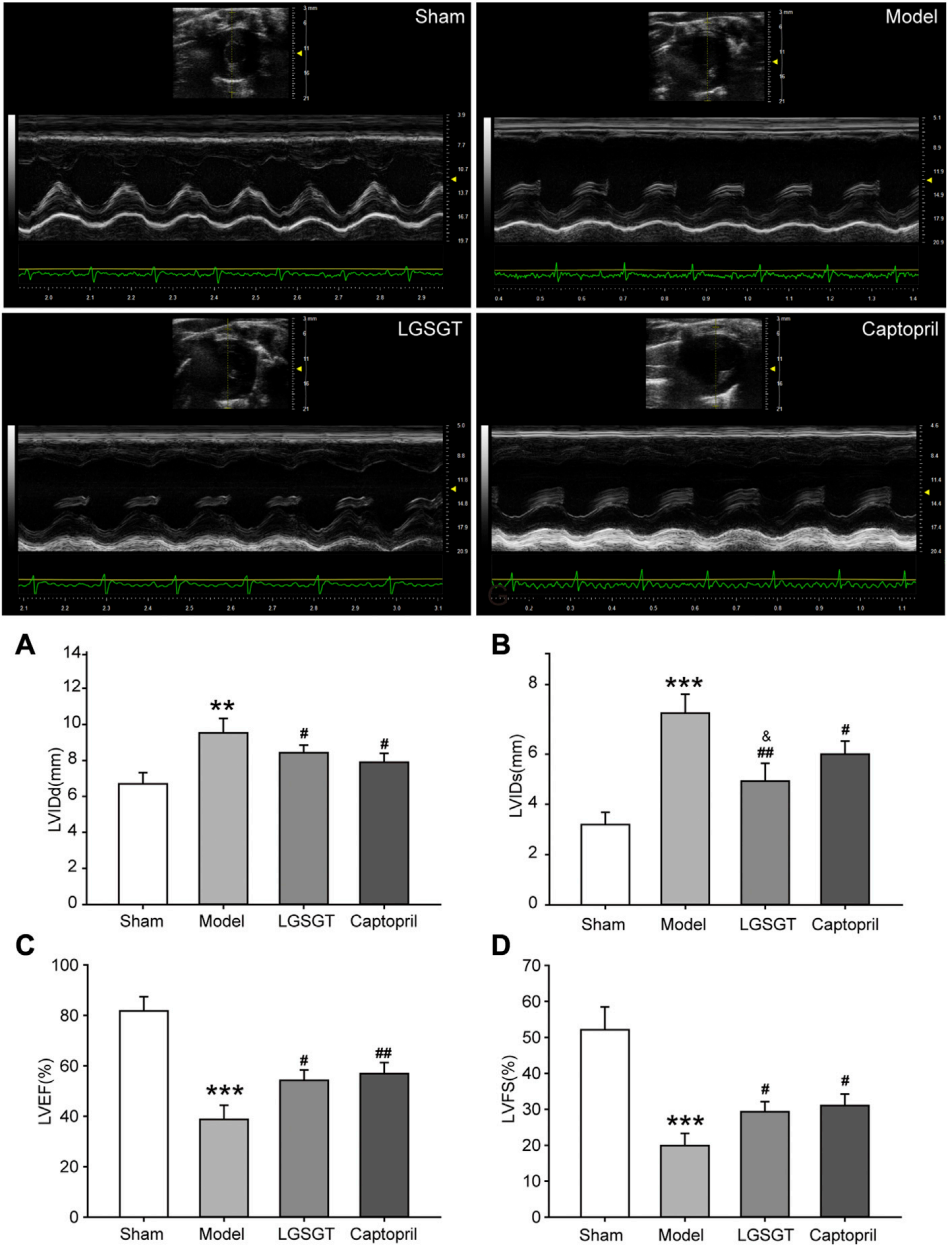
Linggui Zhugan Decoction improves cardiac function in rats with chronic heart failure after myocardial infarction. Ventricular structure and ejection fraction in rats with heart failure measured by echocardiography (*n* = 9) **(A–D)**. *p < 0.05 vs. sham group; **p < 0.01 vs. sham group; ***p < 0.001 vs. sham group; ^#^ *p* < 0.05 vs. model group; ^##^ *p* < 0.01 vs. model group; ^###^ *p* < 0.001 vs. model group.

As shown in Figure 2D, the HW/BW ratio of whole heart weight index in the model group was significantly higher than that in the sham operation group, and the difference was statistically significant (*p* < 0.05). Compared with the model group, the HW/BW ratio of Linggui Zhugan Decoction group and captopril group decreased (*p* < 0.05), and there was no difference between the two groups (*p* > 0.05). This data indicated that Linggui Zhugan Decoction might affect the heart structure, which was also consistent with the result of Wang L. et al. (2020). This data was further verified by echocardiography performed in rats. As shown in Figures 3A–D, after 6 weeks of drug administration, the LVEF% and LVFS% in the model group were significantly lower than those in the sham operation group, and the LVIDd and LVIDs were significantly increased (*p* < 0.01 or *p* < 0.001). Compared with the model group, the LVEF% and LVFS% in the Linggui Zhugan Decoction group and the captopril group were obviously increased, while the LVIDd and LVIDs were apparently decreased (both *p* < 0.05). In the model group, the ventricular systolic and diastolic functions were damaged, and the cardiac function significantly decreased. After treatment with Linggui Zhugan Decoction or captopril, the volume of the ventricular cavity increased, the ventricular remodeling was delayed, and the cardiac function was improved. Moreover, the results of the two-drug regimens showed that LVIDs in the Linggui Zhugan Decoction group were significantly decreased (*p* < 0.05), and there was no significant difference in other ultrasonic indexes. These results indicated that the Linggui Zhugan Decoction group was better than captopril in improving the systolic function of rats, but there was no obvious difference in the effects of the two treatments on cardiac function.

### 3.3 Linggui Zhugan Decoction improves the general state and exercises tolerance of rats with chronic heart failure after myocardial infarction

The sham operation group showed a good mental state, fair activity, smooth and bright hair color, and less struggle. The hair color of rats in the model was dark, while in some, fur was erect and messy. In the early morning, rats engaged in fierce fights accompanied by bites. With the extension of administration time, the fighting gradually eased, the rats became more docile and less irritable, and the overall difference among the groups was no longer obvious.

Cardiac failure can lead to decreased exercise tolerance, which is also a crucial indicator to evaluate the prognosis of heart failure (Passantino et al., 2006). In this study, we used the exhaustive swimming method to examine exercise tolerance. We found that compared with the sham operation group, the exhaustive swimming time of rats in the model group was shortened (*p* < 0.05). Contrary, in the Linggui Zhugan Decoction group and the captopril group, exhaustive swimming time was significantly higher than that in the model group (*p* < 0.05), while there was no difference between the Linggui Zhugan Decoction group and the captopril group (Figure 2C), which indicates that both Linggui Zhugan Decoction and captopril can increase the exercise tolerance of rats with heart failure.

### 3.4 Linggui Zhugan Decoction reduces the level of heart failure markers in rats with heart failure caused by myocardial infarction

**FIGURE 4:**
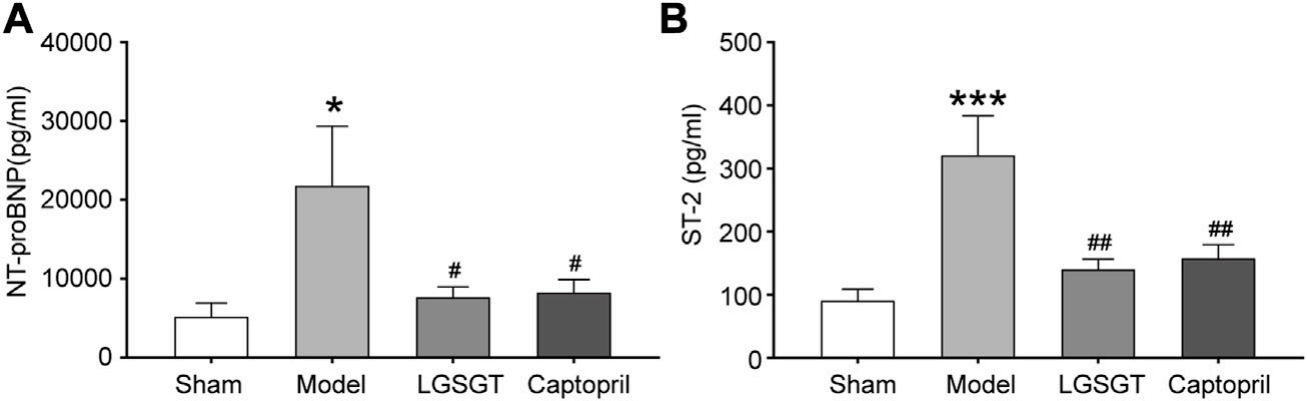
Linggui Zhugan Decoction reduces the level of myocardial markers (NT-proBNP and sST2) in rats with chronic heart failure after myocardial infarction. The levels of biomarker NT-proBNP and sST2 in rats with heart failure detected by ELISA (*n* = 7) **(A, B)**. *p < 0.05 vs. sham group; **p < 0.01 vs. sham group; ***p < 0.001 vs. sham group; ^#^ *p* < 0.05 vs. model group; ^##^ *p* < 0.01 vs. model group; ^###^ *p* < 0.001 vs. model group.

NT-proBNP and sST2 are independent risk factors for heart failure, which have predictive value for the prognosis of heart failure (Pan et al., 2020). In addition, both NT-proBNP and sST2 effect resisting ventricular remodeling (McCarthy and Januzzi, 2018; Daubert et al., 2019). As shown in Figure 4A, we found the serum NT-proBNP level of the model group was significantly increased compared with the sham operation group (*p* < 0.05). Moreover, a significant decrease of the serum NT-proBNP levels was found in the Linggui Zhugan Decoction group and captopril group compared to the model group (*p* < 0.05); there was no significant difference between the Linggui Zhugan Decoction group and captopril group (*p* > 0.05).

As shown in Figure 4B, Similar data were observed for serum ST-2. The ST-2 content in the model group was significantly higher than that in the sham operation group (*p* < 0.001), while the quantification analysis of Serum ST-2 contents within the Linggui Zhugan Decoction group and captopril group demonstrated a significant decrease compared to the model group (*p* < 0.01); there was no significant difference between Linggui Zhugan Decoction group and captopril group (*p* > 0.05). To sum up, our data showed that Linggui Zhugan Decoction could delay myocardial remodeling and improve cardiac function.

### 3.5 Linggui Zhugan decoction reduces mitochondrial injury

**FIGURE 5:**
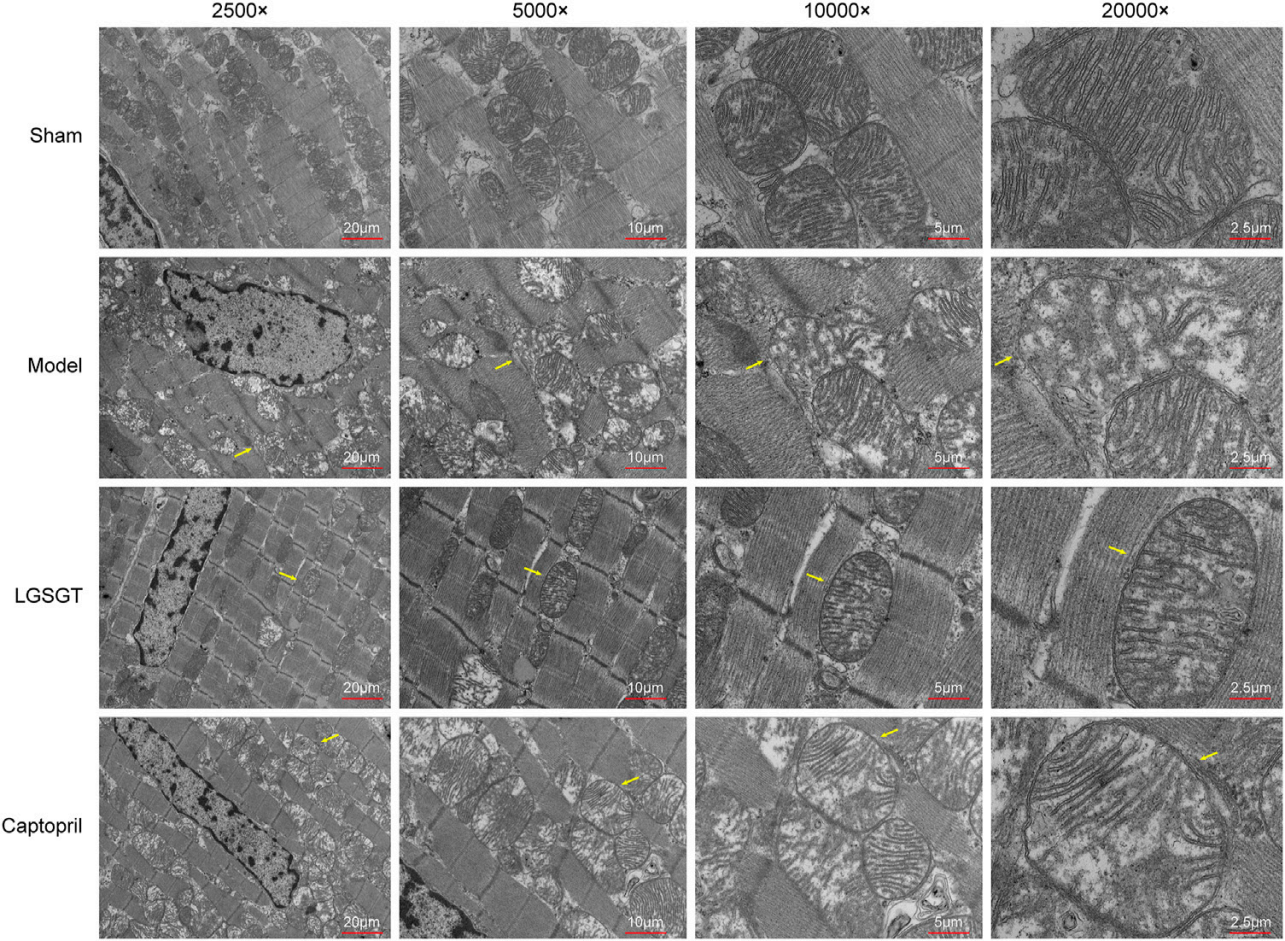
Linggui Zhugan Decoction protects the mitochondria of myocardial cells in rats with heart failure. Observe the differences in the ultrastructure of myocardial cells and mitochondria under the electron microscope.

As shown in Figure 5, TEM was used to assess the structure of mitochondria. In the sham operation group, the cell membrane of myocardial cells was intact; besides, the myocardial fibers with intact sarcomere were neatly arranged, the transverse stripes were clear, and there were many mitochondria between the muscle fibers. Ultrastructural analysis with mitochondria showed that the control group had healthy mitochondria with normal and clear cristae structures. Meanwhile, the internal ridges were clear and closely arranged, and no swelling or vacuoles were found. In contrast, the myocardial structure of model group rats was destroyed, the arrangement of myocardial fibers was loose, disordered, irregular, and even possibly broken. Moreover, the sarcomere was incomplete, transverse striations were not clear, mitochondrial internal cristae were disorderly arranged, the membrane structure was damaged, mitochondria were denatured and swollen, and the outer membrane was incomplete, with vague fusion. Compared to the model group, Linggui Zhugan Decoction or captopril markedly increased integrity in the pathological state of mitochondria (mitochondria of the myocardium were neatly arranged). Transverse stripes of the myocardium with regular sarcomere were slightly blurred, the degree of mitochondrial crista expansion and crista disorder was reduced, and the degree of membrane clarity was increased. This data suggests that Linggui Zhugan Decoction can improve the damage of mitochondrial structure to a certain extent.

**FIGURE 6:**
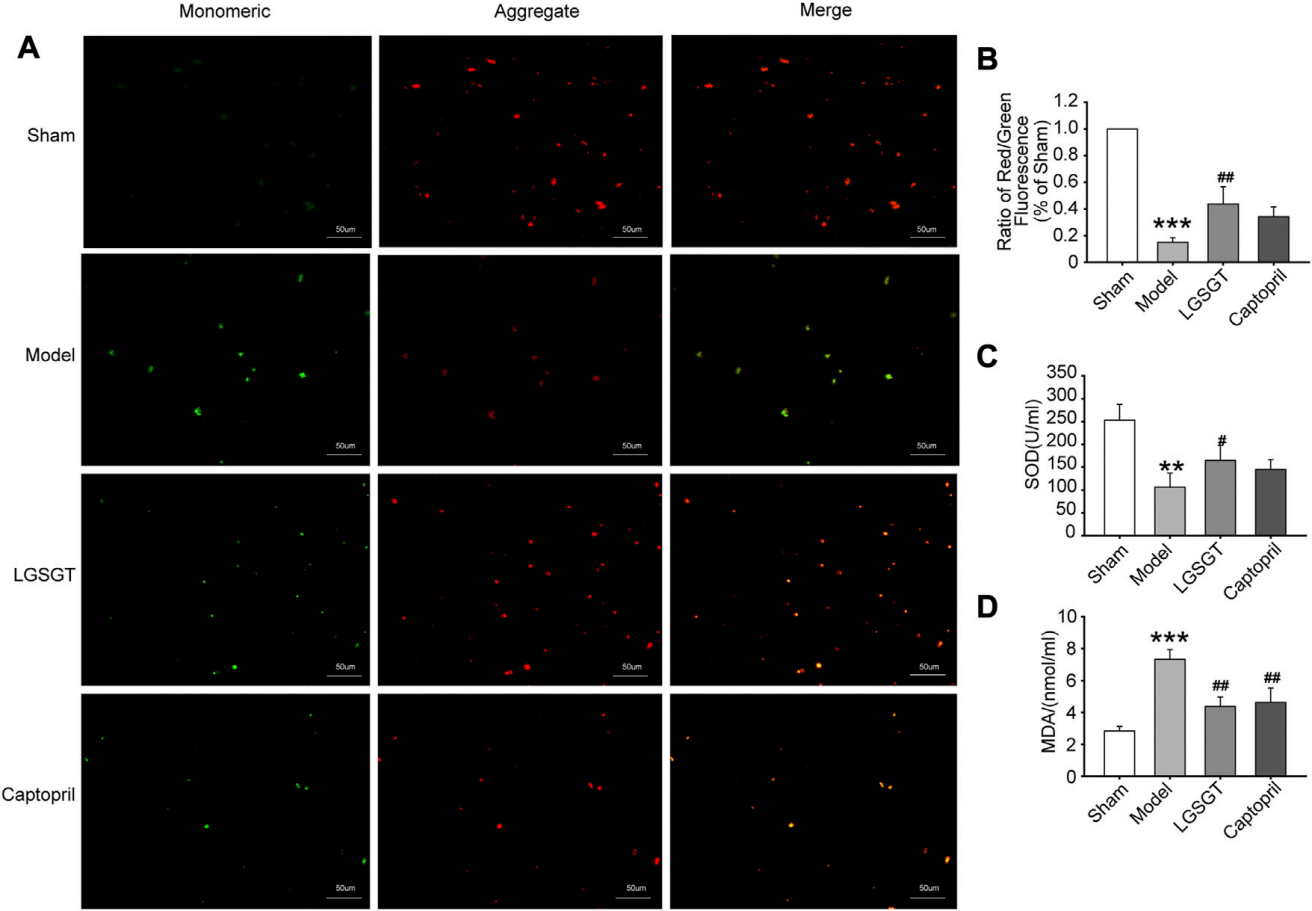
Linggui Zhugan Decoction antagonizes the oxidative stress in rats with heart failure and reduces the mitochondrial damage in myocardial cells. **(A, B)** JC-1 staining was used to observe the effect of Linggui Zhugan Decoction on mitochondrial membrane potential levels (*n* = 5). **(C, D)** ELISA was used to test the effect of Linggui Zhugan Decoction on serum oxidative stress indicators in rats with heart failure (*n* = 7). *p < 0.05 vs. sham group; **p < 0.01 vs. sham group; ***p < 0.001 vs. sham group; ^#^ *p* < 0.05 vs. model group; ^##^ *p* < 0.01 vs. model group; ^###^ *p* < 0.001 vs. model group.

Mitochondrial membrane potential is an electrochemical gradient necessary to maintain the structure and function of mitochondria, which can reflect the functional state of mitochondria and is also an indicator of early myocardial cell apoptosis (Daubert et al., 2019). As shown in Figures 6A, B, we performed mitochondrial staining with JC-1 and found a significant decrease in the membrane potential of myocardial mitochondria in the model group compared to the sham operation group (*p* < 0.001). However, after the intervention of Linggui Zhugan Decoction or captopril intragastrically, the mitochondrial membrane potential in the myocardial tissue of rats in the Linggui Zhugan Decoction group was significantly increased (*p* < 0.01), while that in the captopril group was slightly increased, but the difference was not statistically significant (*p* > 0.05). Therefore, this indicates that Linggui Zhugan Decoction can stabilize myocardial mitochondrial membrane potential in failure.

### 3.6 Linggui Zhugan Decoction alleviates oxidative stress injury in rats with heart failure caused by myocardial infarction

Oxidative stress-induced cardiomyocyte apoptosis is a major cause of heart failure (Zhou et al., 2018), and high levels of oxidative stress markers may reflect the severity of heart failure (Ayoub et al., 2017). As shown in Figures 6C, D, the serum SOD activity was significantly reduced in the model group compared to the sham group (*p* < 0.01). Contrary, SOD activity increased in the Linggui Zhugan Decoction compared to the model group (*p* < 0.05), while there was no difference between the captopril group and model group (*p* > 0.05). Quantification analysis also revealed a distinct increase of the serum MDA content of rats in the model group compared to the sham operation group (*p* < 0.001), while the MDA content in the Linggui Zhugan Decoction group and captopril group was significantly lower than that in the model group (*p* < 0.01). This data suggests that the Linggui Zhugan Decoction group could resist myocardial oxidative damage in rats.

### 3.7 Linggui Zhugan Decoction exerts the protective effect of mitochondria in rats with heart failure by activating the SIRT1-AMPK-PGC1α pathway

**FIGURE 7:**
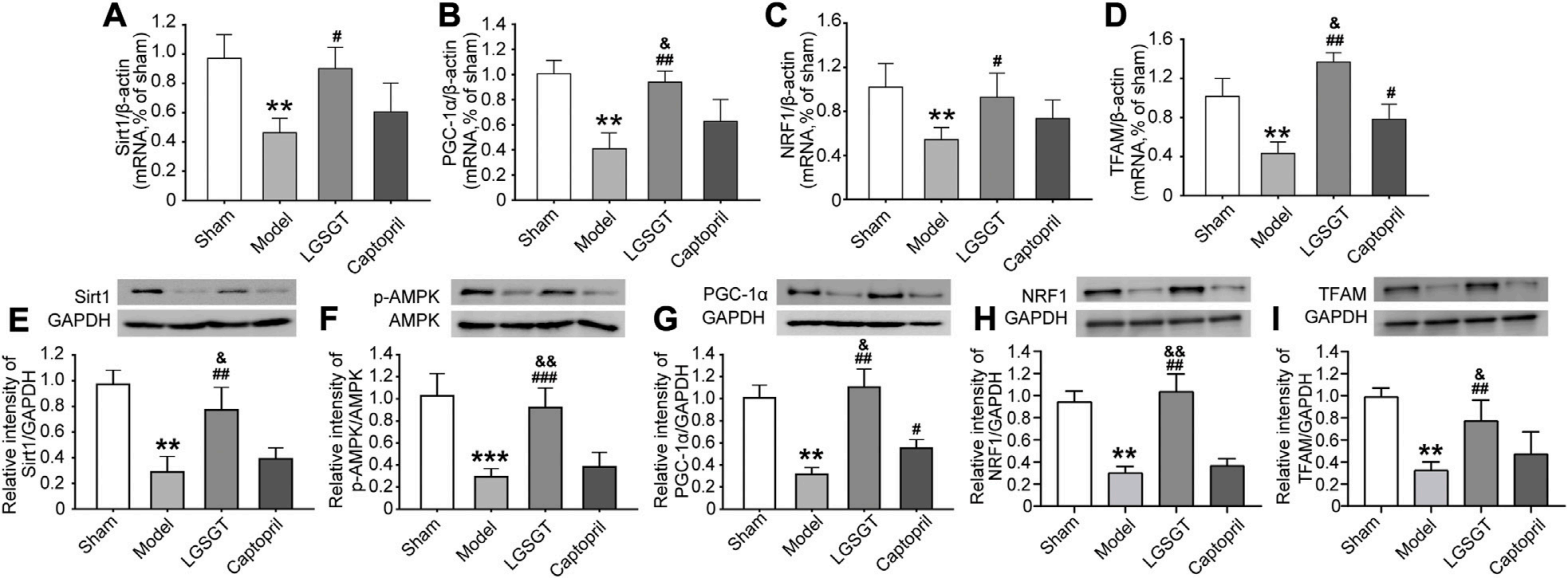
Linggui Zhugan Decoction played a protective role through the SIRT1-AMPK-PGC-1α pathway. **(A–D)** RT-PCR was used to detect the expression levels of the SIRT1-AMPK-PGC-1α pathway and downstream NRF1 and TFAMmRNA in the myocardial tissue of rats with heart failure after treatment with Linggui Zhugan Decoction (*n* = 6). **(E–I)** Western blot was used to detect the expressions of SIRT1, p-AMPK, PGC-1α, NRF1, and TFAM proteins in the myocardial tissue of rats with heart failure after treatment with Linggui Zhugan Decoction (*n* = 3). *p < 0.05 vs. sham group; **p < 0.01 vs. sham group; ***p < 0.001 vs. sham group; ^#^ *p* < 0.05 vs. model group; ^##^ *p* < 0.01 vs. model group; ^###^ *p* < 0.001 vs. model group; ^&^ *p* < 0.05 vs. captopril group; ^&&^ *p* < 0.01 vs. captopril group.

As shown in Figures 7A, B, SIRT1 and PGC-1α mRNA levels in the model group were observably lower than those in the sham operation group, and the differences were statistically significant (*p* < 0.01). Further, compared with the model group, the levels of SIRT1 and PGC-1α mRNA in the Linggui Zhugan Decoction group were increased (*p* < 0.05 or *p* < 0.01). Although the level of the captopril group was higher than that of the model group, the difference was not statistically significant (*p* > 0.05).

In addition, as shown in Figures 7C, D, compared with the model group, the mRNA levels of NRF1 and TFAM in the Linggui Zhugan Decoction group significantly increased (*p* < 0.05). NRF1 and TFAM were the important signaling molecules affecting mitochondrial biogenesis downstream of PGC-1α, indicating that Linggui Zhugan Decoction could increase mitochondrial biogenesis.

As shown in Figures 7E–I, compared with the sham operation group, the expression of SIRT1, p-AMPK, PGC-1α, NRF1 and TFAM protein in the model group was relatively decreased (*p* < 0.01). After the intervention of Linggui Zhugan Decoction, the relative expression of SIRT1, p-AMPK, PGC-1α, NRF1 and TFAM protein was significantly higher compared with the model group (*p* < 0.01), while the relative expression of the p-AMPK protein was higher; yet, the relative expression of AMPK protein did not change, and the difference of p-AMPK/AMPK protein was statistically significant (*p* < 0.05). The SIRT1, p-AMPK/AMPK, NRF1 and TFAM protein levels in captopril-treated rats were similar to those in the model group, but the level of PGC-1α protein was increased (*p* < 0.05).

All of these results indicate that Linggui Zhugan Decoction promotes the activation of AMPK signals in the myocardial tissue of rats with heart failure.

## 4 Discussion

Ischemic cardiomyopathy is one of the common causes of chronic heart failure. Oxidative stress injury associated with decreased ATP production, active oxygen accumulation, and mitochondrial dysfunction has an important role in the occurrence and development of ischemic cardiomyopathy (Akhmedov et al., 2015). The heart is a high-function, high-energy consumption, low-energy storage organ. Failing heart is also sometimes described as an “engine out of fuel” (Neubauer, 2007).

Mitochondria are key organelles in the cell that produce energy. The normal energy metabolism of the myocardium is the material basis for maintaining the stability of the cardiac environment and cardiac systolic and diastolic function of the heart, while the metabolism crisis generated by mitochondria has been associated with myocardial ischemia (Di Lisa et al., 1998). During this condition, metabolites such as reactive oxygen and Ca^2+^ are accumulated in cardiomyocytes in large amounts, resulting in changes in intracellular mitochondrial structure, enhanced activity and function decline of respiratory chain enzymes, decreased energy metabolism and increased oxidative stress, changes in ion concentration gradient inside and outside the mitochondrial membrane, destruction of mitochondrial inner membrane permeability, and unbalance of the mitochondrial energy metabolism process. The opening of mitochondrial membrane conversion pores promotes apoptosis and myocardial remodeling, all of which cause damage to the blood pumping function of the heart, aggravate the load for the maintenance of normal physiological function of the heart, and finally lead to heart failure (Brown et al., 2017). A large number of studies have confirmed that mitochondrial dysfunction and myocardial cell damage caused by oxidative stress are closely related to heart failure (Hsu et al., 2016; Ayoub et al., 2017). In this research, we constructed the rat model of chronic heart failure after myocardial infarction by coronary artery ligation and found that compared with the sham operation group, the rats with heart failure had the poor general condition and lower exercise tolerance, as well as increased levels of heart failure markers ST-2 and NT-proBNP, increased ventricular volume, and decreased ejection fraction. In addition, the serum ROS level and MDA content of rats in the model group also increased, and the cardiomyocytes were in a state of oxidative damage. The pathology and electron microscopy showed that rats with heart failure cardiomyocytes had different morphologies and a disordered arrangement with damaged structure. All this data suggested that rats with heart failure caused by ischemic cardiomyopathy might suffer from oxidative stress, which would induce mitochondrial damage and dysfunction, thus damaging the cardiomyocytes, and, in turn, leading to ventricular remodeling and reducing cardiac function.

Previous studies on the molecular mechanism of Linggui Zhugan Decoction in treating heart failure mainly focused on delaying myocardial remodeling, participating in heart rate regulation, controlling blood pressure, regulating vascular activity, improving glucose metabolism and lipid metabolism (Zhou and Huang, 2020). Over recent years, more and more attention has been paid to the research on the effect of Chinese herbal medicine on improving mitochondrial function. For example, it has been found that Pachymic acid can affect ferroptosis induced by renal ischemia-reperfusion injury in mice, improve mitochondrial outer membrane rupture and internal cristae damage, stabilize mitochondrial ultrastructure, and resist acute kidney injury, which may be related to the activation of NRF2 and the upregulation of the expression of downstream ferroptosis-related proteins GPX4, SLC7A11 and HO-1 (Jiang et al., 2021). Wang et al. (2019) reported that Atractylodes Lactone III acts as an antioxidant by activating the oxidative stress-mediated PI3K/AKT/mTOR pathway, attenuating mitochondrial damage, reducing the accumulation of autophagosomes (AP) and autolysosomes (ALs) in muscle, to resist muscle atrophy. Atractylodes macrocephala Koidz may treat chronic gastritis by improving energy metabolism and regulating inflammatory response (Yang et al., 2019). Gualouguizhi granules can inhibit oxidative damage induced by ischemia-reperfusion in rat brain tissue by activating Nrf2/ARE signaling pathway (Zhang et al., 2018). Glycyrrhizin can protect liver mitochondria from oxidative damage in metabolic syndrome model rats by attenuating mitochondrial oxidative stress and aconitase degradation, improving the activities of electron transport chain-related enzymes (Sil and Chakraborti, 2016). Therefore, we hypothesized that Linggui Zhugan Decoction could treat heart failure by improving mitochondrial function.

In this study, we found that Linggui Zhugan Decoction can improve myocardial cell necrosis of rats with heart failure, improve myocardial fibrosis and alleviate hypertrophy of myocardial cells. At the same time, the levels of heart failure markers and serum ROS and MDA levels were all decreased, and the oxidative stress state was controlled. The mitochondrial function and structure of myocardial cells were normalized, and cardiac function was improved. These results indicate that Linggui Zhugan Decoction can recover mitochondrial membrane potential, reduce mitochondrial damage and myocardial cell oxidative damage, against myocardial fibrosis, and increase the ejection fraction in rats with heart failure.

SIRT1, AMPK, and PGC-1α constitute an energy-sensing system, which has an important role in regulating mitochondrial function. When the ratio of AMP/ATP in cells is increased, the *a* group of Thr172 in AMPK is activated by phosphorylation. p-AMPK can act on a variety of downstream substrates, inhibit the consumption of ATP, and initiate the pathway to generate ATP to maintain the body’s energy metabolism balance, increase the content of intracellular NAD+, and then make NAD + -dependent SIRT1 depleted. Activation and exert its corresponding biological effect (Askin et al., 2020). Disturbance of SIRT1/PGC-1α deacetylation pathway can enhance oxidative stress and promote mitochondrial dysfunction (Wang et al., 2019). Many previous drug studies have confirmed that the SIRT1-AMPK-PGC-1α pathway is the keyway for Chinese herbal medicine to exert its role. Wu et al. (2018) found that icariin could protect mitochondria’s structural and functional integrity by regulating the expression of SIRT1 and reducing the apoptosis induced by MI/R. When the SIRT1 gene was knocked down, or an inhibitor of SIRT1 was administered, the protective effect of icariin could be reversed. Moreover, another study found that naringenin promotes mitochondrial production and relieves mitochondrial oxidative stress by activating AMPK-PGC-1α, thus having a role in resisting MI/R injury (Yu et al., 2019). Melatonin can promote AMPK phosphorylation and upregulation of PGC-1α expression, enhance mitochondria’s oxidative metabolism function, and reduce myocardial apoptosis caused by MI/R (Yu et al., 2017). Moreover, the cardioprotective effects of naringenin and melatonin were significantly reduced when AMPK expression was interfered with, or AMPK inhibitor (Compound C) was administered. Therefore, in order to further explore the possible molecular mechanism of Linggui Zhugan Decoction in the treatment of heart failure from the perspective of mitochondria, we detected the key protein molecules and their mRNA levels. The results showed that the AMPK phosphorylation level of rats with chronic heart failure caused by ischemic heart disease after the intervention of Linggui Zhugan Decoction increased, while the protein and mRNA levels of SIRT1 and PGC-1α were significantly increased. This indicated that Linggui Zhugan Decoction could activate the SIRT1/AMPK/PGC-1α pathway to protect mitochondria and resist oxidative stress in myocardial cells.

PGC-1α is a key factor in regulating mitochondrial biosynthesis (Sharma et al., 2013). PGC-1α activates NRF-1 and NRF-2 and increases the transcription of several mitochondrial DNA genes, thus participating in the process of the respiratory chain, which is especially more significant in the cardiovascular system (Whitaker et al., 2016). Cardiovascular cells such as myocardium and vascular endothelium regulate mitochondrial production through the PGC-1α signaling pathway, thereby maintaining and repairing the cell structure and function. Similarly, in this experiment, we found that Linggui Zhugan Decoction increased the expression of NRF1 and TFAM mRNA and protein downstream of PGC-1α and increased mtDNA content in rats with heart compared with the model group. We also found that, although captopril could repair mitochondrial damage in myocardial cells of rats with heart failure, it did not affect the protein and mRNA levels of SIRT1, p-AMPK, and PGC-1α. Previous studies have confirmed the protective effect of captopril on myocardial cells (Mujkosová et al., 2010). A previous study suggested that captopril has a protective role by improving energy metabolism (Gvozdjáková et al., 1999) and weakening apoptosis (Lin et al., 2013). Yet, there is insufficient evidence supporting that captopril can affect the SIRT1-AMPK-PGC-1α pathway.

In conclusion, our *in vivo* findings showed that the SIRT1-AMPK-PGC-1α signaling pathway was inhibited during heart failure, which is consistent with previous studies. Furthermore, the results showed that Linggui Zhugan Decoction could activate the SIRT1-AMPK-PGC-1α signaling pathway, restore mitochondrial membrane potential, reduce the degree of mitochondrial damage and oxidative damage, promote mitochondrial biogenesis, and improve cardiac function. These results indicated that SIRT1-AMPK-PGC-1α is the key signaling pathway mediating the protection of Linggui Zhugan Decoction on the mitochondrial function in rats with heart failure.

Although this experiment verified the protective effect of Linggui Zhugan Decoction on myocardial mitochondrial function in rats with heart failure, reasonable treatment-related dose ranges and dose-response relationship cannot be clearly defined due to the lack of positive drug multiple-dose comparisons in the experimental design. Furthermore, the effects of Linggui Zhugan Decoction on mitochondrial oxygen consumption and oxidative phosphorylation, and which effective compounds in decoction have an effect on mitochondria, need to be further explored.

## Data Availability

The original contributions presented in the study are included in the article/Supplementary Material, further inquiries can be directed to the corresponding authors.
