# Supplementary figures and images for: Linggui Zhugan Decoction activates the SIRT1-AMPK-PGC1α signaling pathway to improve mitochondrial and oxidative damage in rats with chronic heart failure caused by myocardial infarction
Source: Front Pharmacol. 2023 Apr 5;14:1074837. doi: 10.3389/fphar.2023.1074837 (PMC10113531; doi:10.3389/fphar.2023.1074837)

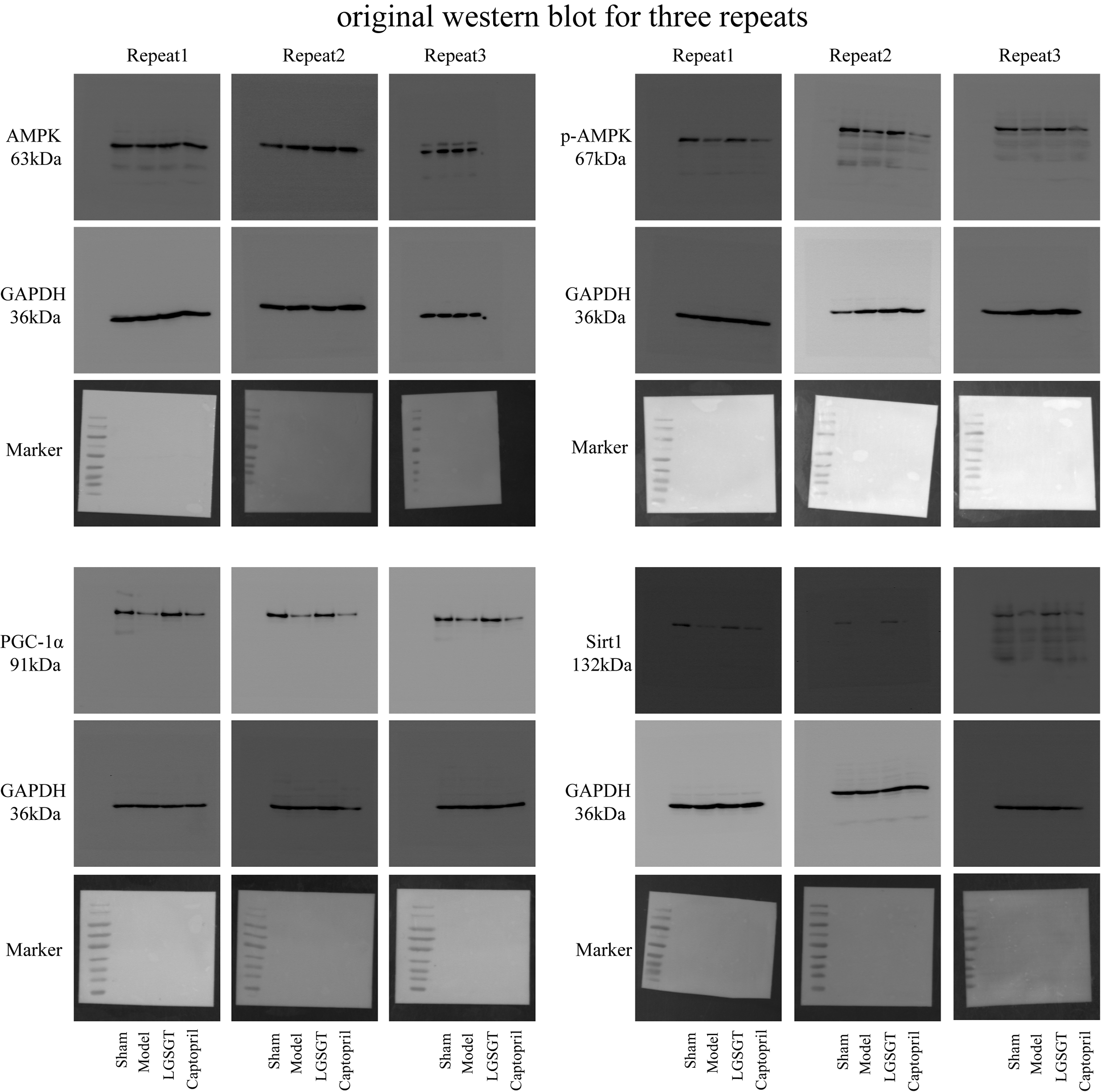

Supplement: Supplementary file 1 [file Image1.TIF]
