# Supplementary material for: Linggui Zhugan Decoction activates the SIRT1-AMPK-PGC1α signaling pathway to improve mitochondrial and oxidative damage in rats with chronic heart failure caused by myocardial infarction
Source: Front Pharmacol. 2023 Apr 5;14:1074837. doi: 10.3389/fphar.2023.1074837 (PMC10113531; doi:10.3389/fphar.2023.1074837)
Supplement: Supplementary file 2 [file DataSheet1.PDF]

Figure 1

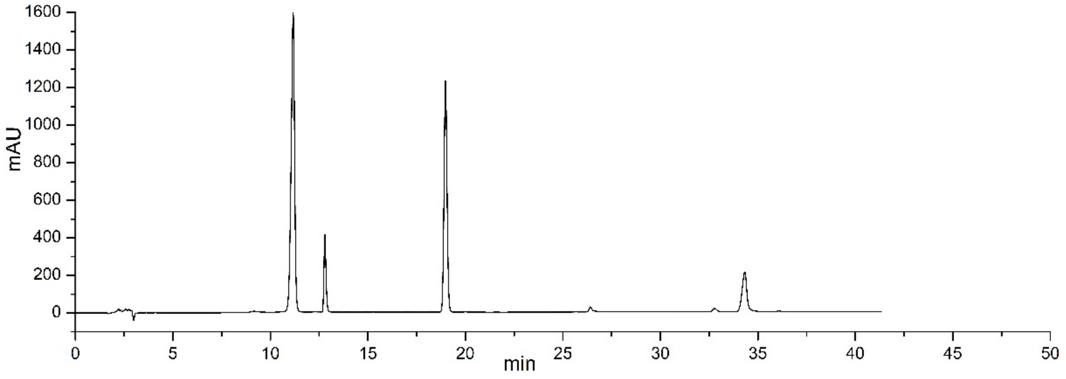

mAU
1600
1400
1200
1000
800
600
400
200
0
0
5
10
15
20
25
30
35
40
45
50
min

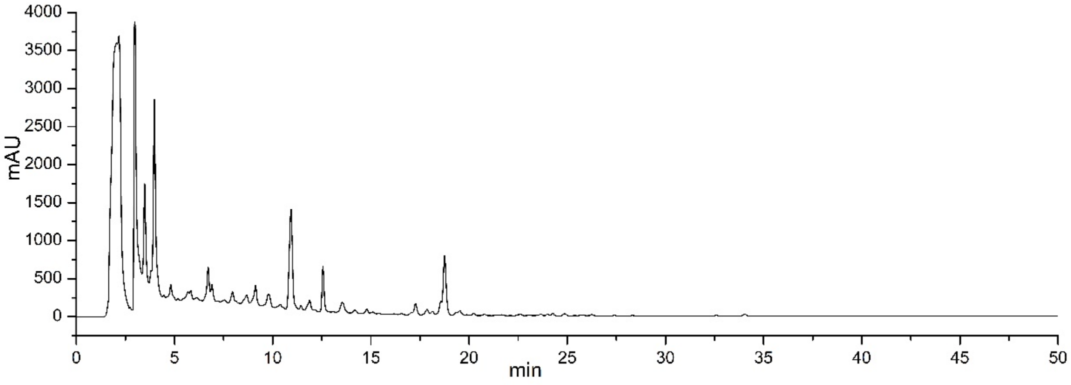

mAU
4000
3500
3000
2500
2000
1500
1000
500
0
0
5
10
15
20
25
30
35
40
45
50
min

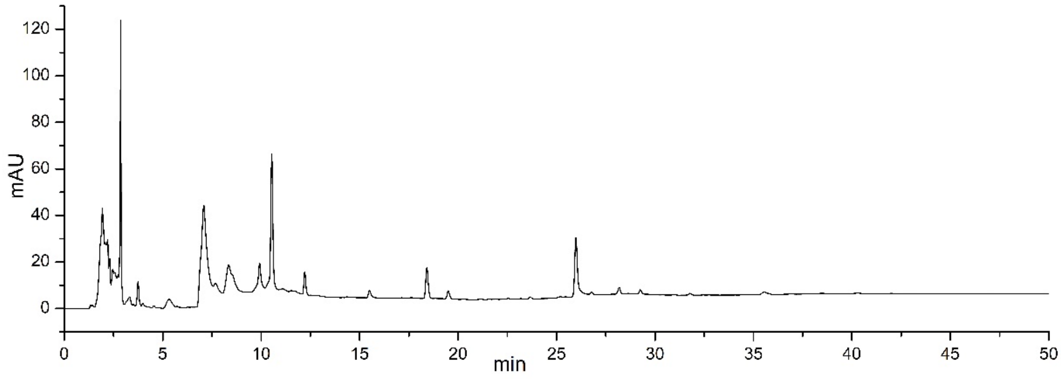

	Standards			LGSGT			serum			
	Retention time	Peak area	Standard concentration	Retention time	Peak area	Active ingredient concentration	Retention time	Peak area	Serum concentration after dilution	The actual serum concentration of each substance
cinnamic acid	11.169	19946.03	204.6	10.934	14963.61	153.4919732	10.539	407.6701	4.181750098	12.5452503
glycyrrhizic acid	12.793	3165.66	435.8	12.557	4513.441	621.3420582	12.211	65.5447	9.023198751	27.06959625
atractylenolide III	18.979	13317.45	102.2	18.753	8791.831	67.46975086	18.414	126.0993	0.967703832	2.903111496
Pachymic acid	34.329	3749.73	184.5	34.038	379.5663	18.67600514				

Figure 2

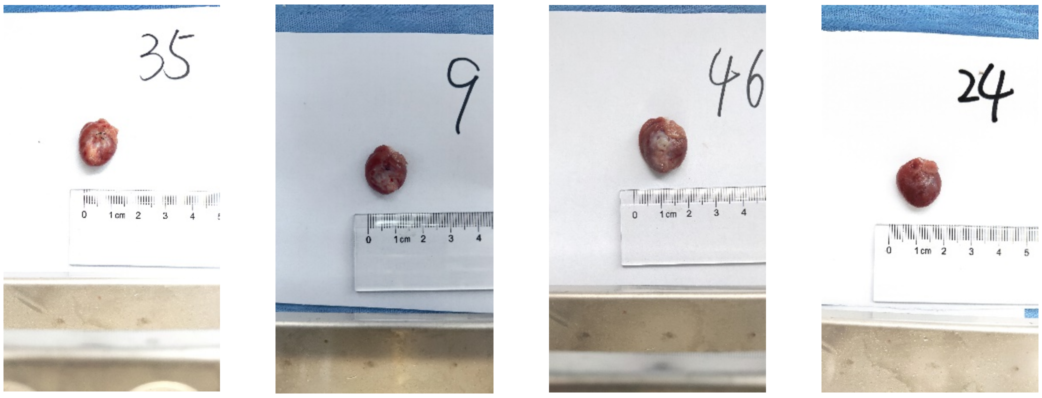

Captopril LGSGT model sham

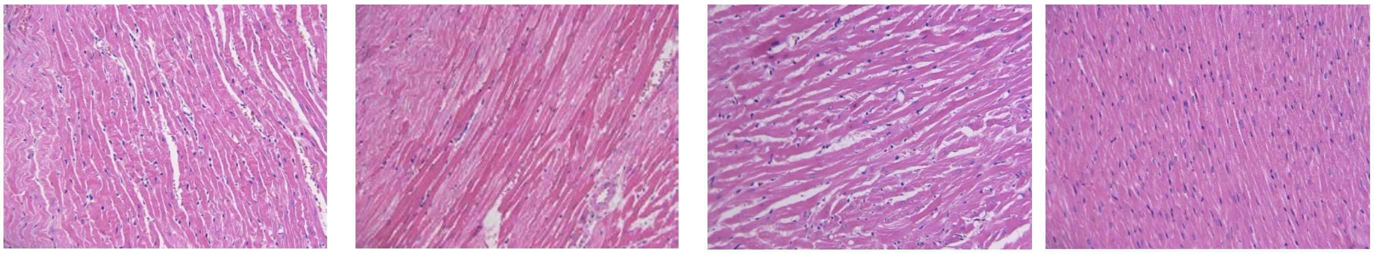

Captopril LGSGT model Sham

Group	Number	BW	HW	HW/BW	exhausted swimming time (s)
LGSGT	6	435	1.151	2.646	1047
	13	463	1.259	2.7192	1185
	19	470	1.302	2.7702	956
	9	478	1.323	2.7678	1069
	67	442	1.176	2.6606	1243
	60	449	1.187	2.6437	1362
	23	490	1.312	2.6776	1270
	51	454	1.171	2.5793	1439
	10	459	1.183	2.5773	1333
	8	495	1.235	2.4949	1549
captopril	41	456	1.208	2.6491	1473
	35	469	1.191	2.5394	1360
	11	480	1.242	2.5875	1601
	42	496	1.283	2.5867	1371
	33	495	1.259	2.5434	893
	34	480	1.256	2.6167	1347
	3	464	1.186	2.556	1094
	2	460	1.214	2.6391	1116
	30	500	1.348	2.696	1242
	37	478	1.234	2.5816	1078
model	45	456	1.238	2.7149	644
	57	441	1.226	2.78	751
	44	436	1.196	2.7431	737
	29	448	1.218	2.7188	1037
	7	436	1.285	2.9472	834
	25	425	1.224	2.88	694
	66	436	1.232	2.8257	748
	49	433	1.194	2.7575	893
	46	548	1.396	2.5474	925
sham	12	522	1.302	2.4943	1760
	24	424	1.107	2.6108	1957
	28	468	1.128	2.4103	1524
	38	470	1.167	2.483	1635
	1	485	1.345	2.7732	1560
	39	488	1.275	2.6127	1536
	65	501	1.375	2.7445	1447
	68	459	1.169	2.5468	1484
	52	467	1.141	2.4433	1837
	59	524	1.356	2.5878	1749
	58	560	1.411	2.5196	1068

Figure 3

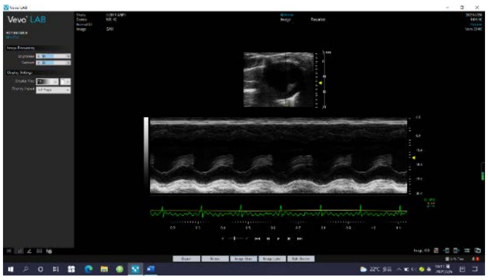

Captopril

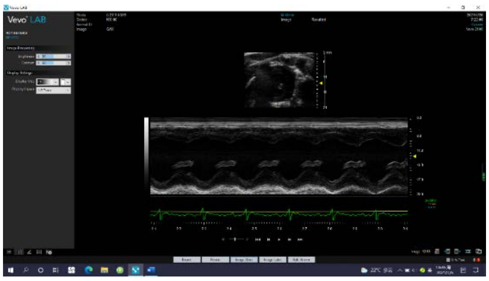

LGSGT

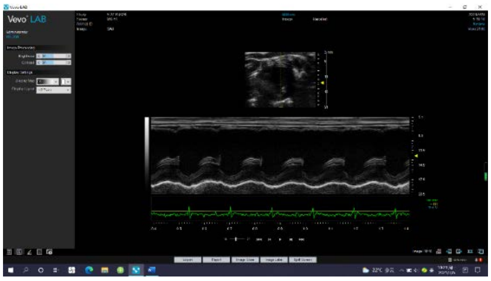

Model

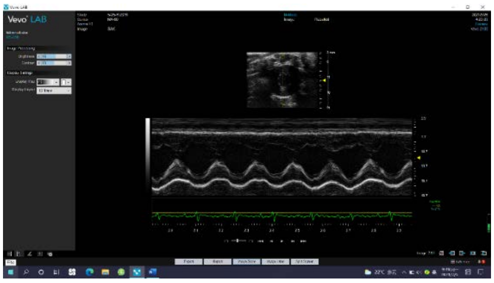

Sham

Group	number	EF	FS	LVIDd	LVIDs
model	44	33.2623	16.6902	9.62983	8.02259
	66	39.0611	19.9708	9.34379	7.47776
	49	39.3255	20.1439	9.46638	7.55948
	46	52.3564	28.1931	9.05679	6.50339
	57	33.7025	16.8224	8.74448	7.27345
	45	35.9032	18.1306	9.23053	7.55697
	29	39.125	20.0191	9.41716	7.53193
	7	39.1711	20.0194	9.23761	7.3883
	25	37.3419	19.2397	11.5717	9.34534
sham	24	86.0441	56.457	6.27842	2.73382
	59	76.384	45.6075	5.56118	3.02487
	58	78.1327	47.4157	5.78207	3.04046
	28	84.7633	55.2786	7.08921	3.1704
	39	71.482	42	7.27632	4.22026
	65	89.1841	61.1594	7.17237	2.78579
	68	89.3472	61.3933	7.16197	2.765
	38	80.4094	50.4301	7.20294	3.57049
	1	80.0732	50	7.02684	3.51342
	52	77.9658	47.839	6.97487	3.63816
	12	85.7145	55.9524	6.11211	2.69224
captopril	42	65.3712	37.3266	8.24303	5.16618
	34	52.1556	27.6353	7.29711	5.28053
	2	60.5209	33.3333	7.14118	4.76079
	37	57.6351	31.3725	7.42184	5.09342
	11	60.3891	33.6574	8.55487	5.67553
	41	56.612	30.8579	8.11829	5.61316
	35	52.6524	28.0438	7.59855	5.46763
	33	57.5287	31.5051	8.14947	5.58197
	3	55.2399	29.9751	8.35737	5.85224
	30	50.9141	27.027	8.07671	5.89382
LGSGT	67	56.079	30.4117	7.82724	5.44684
	9	60.0392	33.3333	8.295	5.53
	8	60.5767	33.7469	8.37816	5.55079
	19	57.1883	31.3505	8.47207	5.81603
	6	53.1345	28.5171	8.20145	5.86263
	13	47.9249	25.1996	8.46305	6.3304
	60	53.8399	28.9575	8.07671	5.7379
	23	50.626	26.8695	8.20145	5.99776
	51	51.3097	27.2844	8.07671	5.87303
	10	51.6533	27.5776	8.36776	6.06013

Figure 4

ST-2

LGSGT	6	0.429	139.603
	13	0.392	128.035
	19	0.456	148.175
	9	0.461	149.774
	67	0.565	183.888
	60	0.414	134.889
	23	0.568	184.896
	51	0.615	200.865
	10	0.335	110.614
	8	0.498	161.725
captopril	41	0.487	158.151
	35	0.49	159.124
	11	0.521	169.257
	42	0.51	165.645
	33	0.578	188.266
	34	0.455	147.855
	3	0.446	144.987
	2	0.352	115.759
	30	0.54	175.539
	37	0.619	202.24
model	45	0.731	241.695
	57	0.851	286.055
	44	0.988	339.335
	29	0.735	243.139
	7	0.97	332.174
	25	0.899	304.402
	66	1.259	453.011
	49	0.837	280.768
	46	0.925	314.485
sham	12	0.229	79.5127
	24	0.194	69.6129
	28	0.323	107.009
	38	0.386	126.178
	1	0.248	84.9637
	39	0.316	104.916
	65	0.259	88.1443
	68	0.214	75.2475
	52	0.208	73.5508
	59	0.368	120.641
	58	0.374	122.481

NT-proBNP

		450 1	540 1		y = 0.0002x + 0.0372		
LGSGT	6	0.225	0.048		0.177	699	6990
	13	0.198	0.049		0.149	559	5590
	19	0.362	0.05		0.312	1374	13740
	9	0.295	0.046		0.249	1059	10590
	67	0.251	0.048		0.203	829	8290
	60	0.317	0.046		0.271	1169	11690
	23	0.418	0.048		0.37	1664	16640
	51	0.23	0.045		0.185	739	7390
	10	0.22	0.046		0.174	684	6840
	8	0.224	0.044		0.18	714	7140
captopril	41	0.286	0.044		0.242	1024	10240
	35	0.297	0.045		0.252	1074	10740
	11	0.253	0.045		0.208	854	8540
	42	0.332	0.051		0.281	1219	12190
	33	0.585	0.052		0.533	2479	24790
	34	0.208	0.046		0.162	624	6240
	3	0.286	0.049		0.237	999	9990
	2	0.201	0.046		0.155	589	5890
	30	0.231	0.045		0.186	744	7440
	37	0.257	0.044		0.213	879	8790
model	45	0.623	0.053		0.57	2664	26640
	57	0.415	0.044		0.371	1669	16690
	44	0.79	0.052		0.738	3504	35040
	29	0.308	0.048		0.26	1114	11140
	7	0.296	0.051		0.245	1039	10390
	25	0.474	0.049		0.425	1939	19390
	66	0.363	0.047		0.316	1394	13940
	49	0.587	0.049		0.538	2504	25040
	46	0.379	0.044		0.335	1489	14890
sham	12	0.232	0.045		0.187	749	7490
	24	0.396	0.057		0.339	1509	15090
	28	0.194	0.045		0.149	559	5590
	38	0.169	0.044		0.125	439	4390
	1	0.213	0.047		0.166	644	6440
	39	0.347	0.055		0.292	1274	12740
	65	0.198	0.045		0.153	579	5790
	68	0.16	0.045		0.115	389	3890
	52	0.213	0.045		0.168	654	6540
	59	0.127	0.048		0.079	209	2090
	58	0.148	0.046		0.102	324	3240

Figure 5

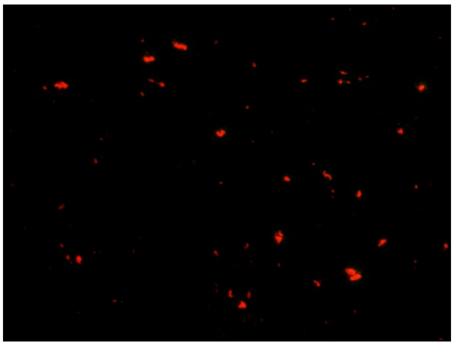
sham merge

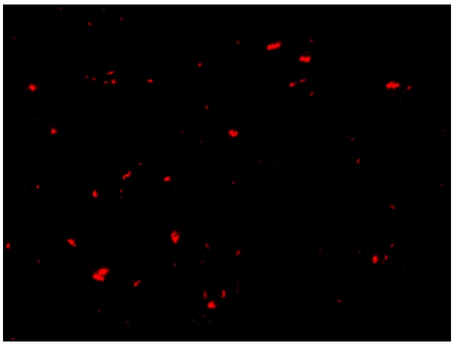
sham aggregate

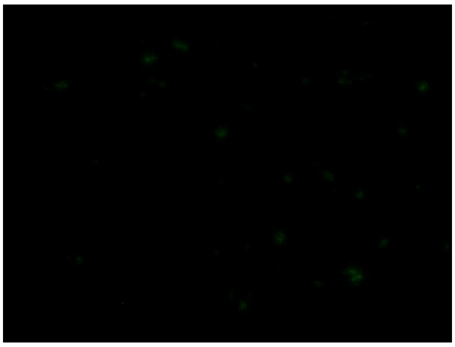
sham monomerice

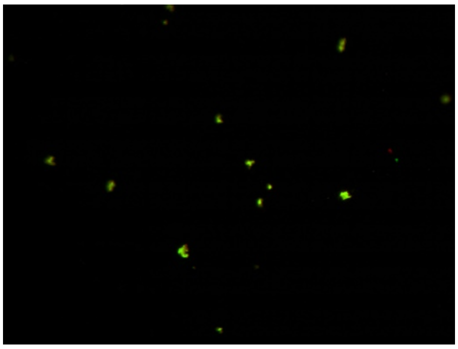

model merge

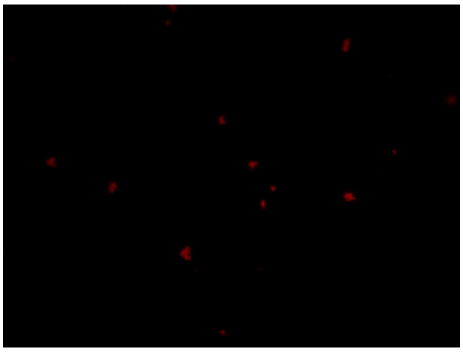

model aggregate

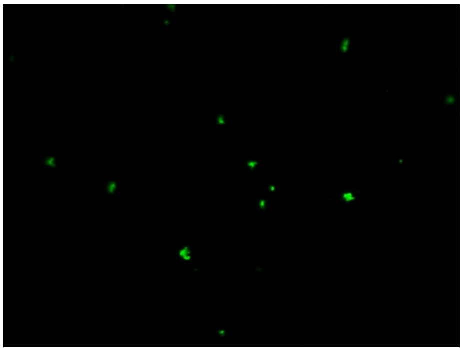

model monomerice

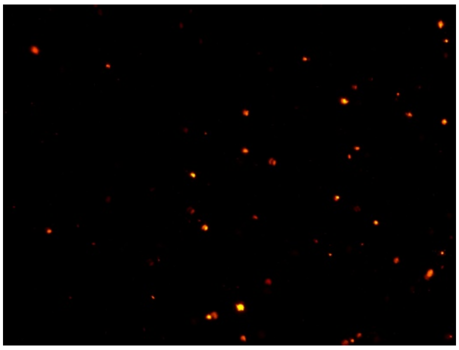

LGSGT merge

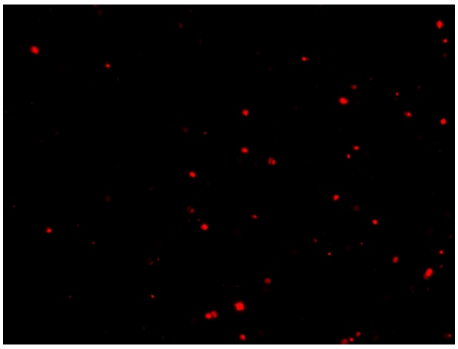

LGSGT aggregate

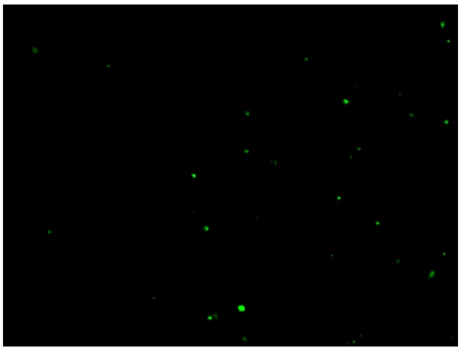

LGSGT monomerice

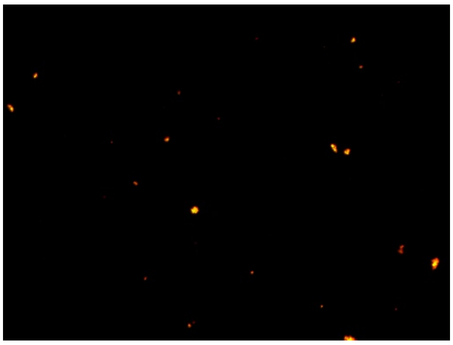

captopril merge

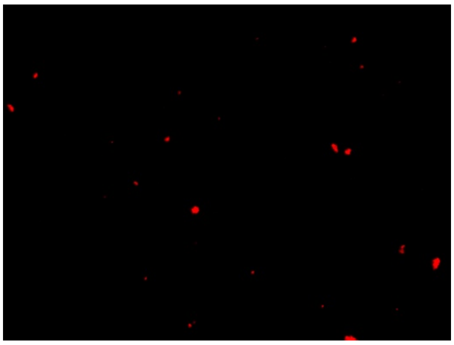

captopril aggregate

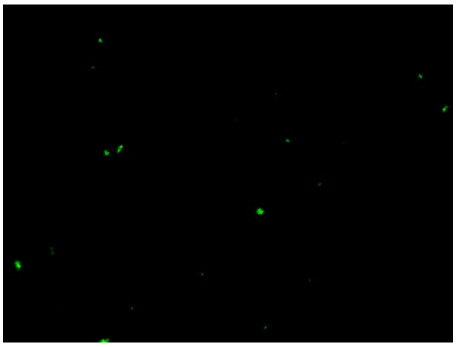

captopril monomerice

mtDNA

		β-actin	mtDNA
LGSGT	6	21.59	22.19
	13	21.27	19.41
	19	20.37	18.78
	9	21.59	20.04
	67	21.56	18.89
	60	21.6	17.86
	23	21.56	18.44
	51	21.35	18.89
	10	21.56	17.6
	8	22.51	21.36
captopril	41	23.17	19.45
	35	21.1	19.17
	11	20.53	18.09
	42	22.63	20.63
	33	22.18	20.22
	34	21.88	17.54
	3	22.79	21.23
	2	21.8	20.07
	30	22.16	18.25
	37	20.99	21.01

model	45	20.31	20.4
	57	20.92	20.04
	44	21.76	20.21
	29	21.87	23.02
	7	21.28	22.14
	25	20.42	22.14
	66	22.3	17.74
	49	22.69	18.11
	46	21.43	20.03
sham	12	21.97	18.3
	24	22.1	22.67
	28	20.9	19.09
	38	23.57	22.37
	1	21.28	17.91
	39	21.07	19.21
	65	21.22	20.2
	68	22.49	19.42
	52	21.25	18.87
	59	20.02	18.01
	58	21.32	19.61

SOD

							SOD
captopril	2	0.258	0.063	0.195	0.3	0.65	117.6
	33	0.196	0.063	0.133	0.3	0.44333	187.04
	37	0.21	0.063	0.147	0.3	0.49	171.36
	30	0.149	0.063	0.086	0.3	0.28667	239.68
	41	0.216	0.063	0.153	0.3	0.51	164.64
	35	0.223	0.063	0.16	0.3	0.53333	156.8
	11	0.194	0.063	0.131	0.3	0.43667	189.28
	42	0.257	0.063	0.194	0.3	0.64667	118.72
	3	0.228	0.063	0.165	0.3	0.55	151.2
	34	0.246	0.063	0.183	0.3	0.61	131.04
LGSGT	51	0.251	0.063	0.188	0.3	0.62667	125.44
	23	0.231	0.063	0.168	0.3	0.56	147.84
	8	0.272	0.063	0.209	0.3	0.69667	101.92
	10	0.189	0.063	0.126	0.3	0.42	194.88
	60	0.204	0.063	0.141	0.3	0.47	178.08
	9	0.229	0.063	0.166	0.3	0.55333	150.08
	13	0.183	0.063	0.12	0.3	0.4	201.6
	19	0.198	0.063	0.135	0.3	0.45	184.8
	6	0.213	0.063	0.15	0.3	0.5	168
	67	0.189	0.063	0.126	0.3	0.42	194.88
model	29	0.297	0.063	0.234	0.3	0.78	73.92
	44	0.273	0.063	0.21	0.3	0.7	100.8
	49	0.193	0.063	0.13	0.3	0.43333	190.4
	7	0.182	0.063	0.119	0.3	0.39667	202.72
	46	0.254	0.063	0.191	0.3	0.63667	122.08
	45	0.228	0.063	0.165	0.3	0.55	151.2
	25	0.276	0.063	0.213	0.3	0.71	97.44
	57	0.246	0.063	0.183	0.3	0.61	131.04
	66	0.303	0.063	0.24	0.3	0.8	67.2
sham	39	0.185	0.063	0.122	0.3	0.40667	199.36
	24	0.173	0.063	0.11	0.3	0.36667	212.8
	38	0.126	0.063	0.063	0.3	0.21	265.44
	1	0.097	0.063	0.034	0.3	0.11333	297.92
	68	0.263	0.063	0.2	0.3	0.66667	112
	59	0.18	0.063	0.117	0.3	0.39	204.96
	28	0.146	0.063	0.083	0.3	0.27667	243.04
	12	0.109	0.063	0.046	0.3	0.15333	284.48
	58	0.206	0.063	0.143	0.3	0.47667	175.84
	65	0.131	0.063	0.068	0.3	0.22667	259.84
	52	0.051	0.063	-0.012	0.3	-0.04	349.44

MDA

					MDA
LGSGT	51	0.071	0.044	0.027	7.10526
	23	0.063	0.044	0.019	5
	8	0.064	0.044	0.02	5.26316
	10	0.062	0.044	0.018	4.73684
	60	0.059	0.044	0.015	3.94737
	9	0.061	0.044	0.017	4.47368
	13	0.058	0.044	0.014	3.68421
	19	0.06	0.044	0.016	4.21053
	6	0.068	0.044	0.024	6.31579
	67	0.058	0.044	0.014	3.68421
captopril	2	0.059	0.044	0.015	3.94737
	33	0.072	0.044	0.028	7.36842
	37	0.059	0.044	0.015	3.94737
	30	0.062	0.044	0.018	4.73684
	41	0.064	0.044	0.02	5.26316
	35	0.062	0.044	0.018	4.73684
	11	0.07	0.044	0.026	6.84211
	42	0.065	0.044	0.021	5.52632
	3	0.056	0.044	0.012	3.15789
	34	0.066	0.044	0.022	5.78947
sham	39	0.053	0.044	0.009	2.36842
	24	0.064	0.044	0.02	5.26316
	38	0.054	0.044	0.01	2.63158
	1	0.056	0.044	0.012	3.15789
	68	0.054	0.044	0.01	2.63158
	59	0.055	0.044	0.011	2.89474
	28	0.066	0.044	0.022	5.78947
	12	0.056	0.044	0.012	3.15789
	58	0.055	0.044	0.011	2.89474
	65	0.056	0.044	0.012	3.15789
	52	0.054	0.044	0.01	2.63158
model	29	0.069	0.044	0.025	6.57895
	44	0.072	0.044	0.028	7.36842
	49	0.053	0.044	0.009	2.36842
	7	0.073	0.044	0.029	7.63158
	46	0.069	0.044	0.025	6.57895
	45	0.071	0.044	0.027	7.10526
	25	0.065	0.044	0.021	5.52632
	57	0.075	0.044	0.031	8.15789
	66	0.074	0.044	0.03	7.89474

Figure 6

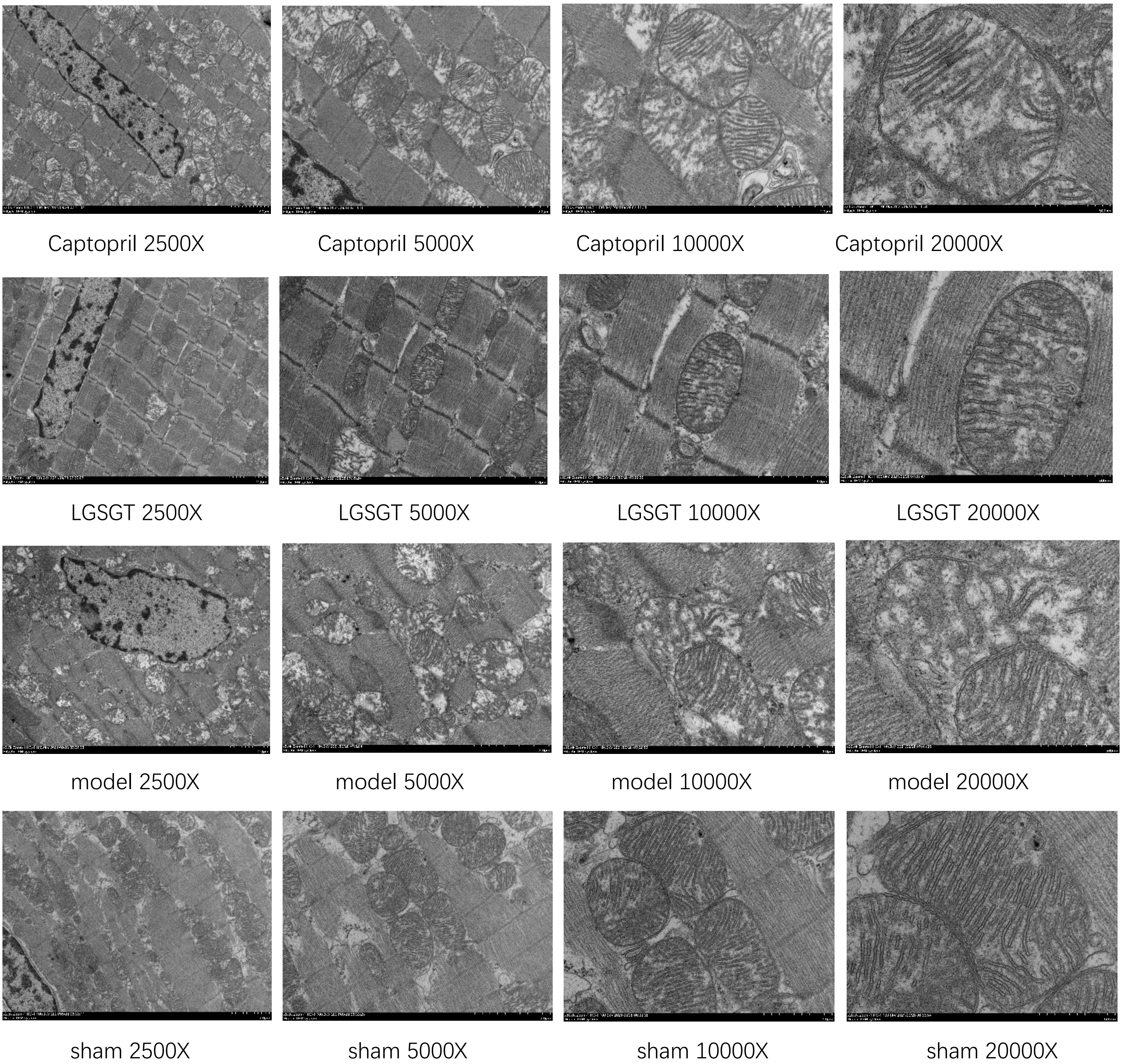
Captopril 2500X
Captopril 5000X
Captopril 10000X
Captopril 20000X
LGSGT 2500X
LGSGT 5000X
LGSGT 10000X
LGSGT 20000X
model 2500X
model 5000X
model 10000X
model 20000X
sham 2500X
sham 5000X
sham 10000X
sham 20000X

Figure 7

		β-actin	Sirt1	AMPK	PGC-1a	TFAM	NRF
	6	21.59	30.47	NoCt	28.62	24.62	28.36
	13	21.27	27.73	24.58	26.3	22.08	21.53
	19	20.37	28.67	23.88	27.69	22.27	21.9
	9	21.59	29.29	23.76	30.21	23.73	23.5
LGSGT	67	21.56	28.84	24.03	30.12	23.72	20.92
	60	21.6	28	25.45	30.32	22.84	23.68
	23	21.56	29.23	24.68	30.45	23.66	23.28
	51	21.35	29.75	27.51	29.87	23.55	23.29
	10	21.56	29.34	25.54	32.28	24.82	23.86
	8	22.51	29.61	23.57	31	24.61	23.86
	41	23.17	30.9	26.95	32.32	28.89	26.01
	35	21.1	27.51	24.99	28.43	24.02	24.53
	11	20.53	29.69	27.34	30.41	23.02	20.15
	42	22.63	30.93	24.24	31.8	25.76	27.54
captopril	33	22.18	30.03	24.06	32.51	25.01	24.62
	34	21.88	29.86	25.39	30.04	23.5	23.07
	3	22.79	30.64	24.51	33.68	35.28	24.81
	2	21.8	28.28	25.18	30.59	24.57	22.07
	30	22.16	26.55	25.84	31.32	25.37	24.95
	37	20.99	26.74	24.72	30.49	22.1	22.93
	45	20.31	28.51	22.91	27	21.94	23.44
	57	20.92	28.36	24.8	30.51	25.33	21.19
	44	21.76	29.09	22.15	32.25	31.78	21.17
	29	21.87	30.51	25.54	31.51	23.77	24.74
model	7	21.28	29.82	30.04	31.88	24.76	23.68
	25	20.42	29.18	23.7	29.77	23.93	26.36
	66	22.3	30.35	28.91	31.49	25.91	21.54
	49	22.69	29.28	26.26	28.82	26.51	25.51
	46	21.43	30.76	25.26	31.55	28.14	23.85
	12	21.97	29.1	24.69	30.56	24.6	21.74
	24	22.1	30.25	25.81	30.85	24.92	27.38
	28	20.9	28.68	24.48	30.4	23.27	22.82
	38	23.57	29.74	27.97	32.04	25.28	24.22
	1	21.28	28.46	24.78	26.85	24.71	21.88
sham	39	21.07	28.68	22.71	29.45	23.53	22.47
	65	21.22	27.73	24.79	29.84	23.73	22.94
	68	22.49	29.81	26.18	31.05	24.7	24.17
	52	21.25	28.63	26.62	31.17	23.91	23.16
	59	20.02	27.13	24.11	28.58	20.55	22.78
	58	21.32	29.27	24.76	30.9	25.98	23.61

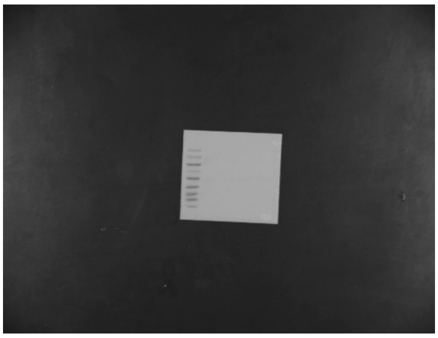
A AMPK marker

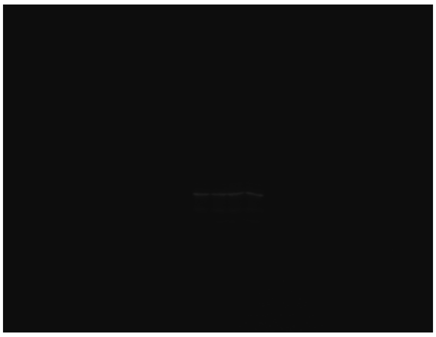
A AMPK

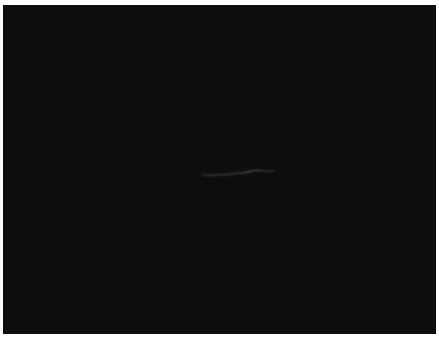
A AMPK GAPDH

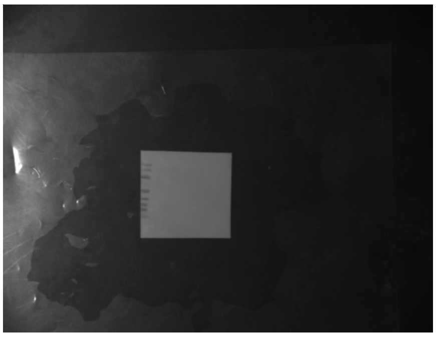

B AMPK marker

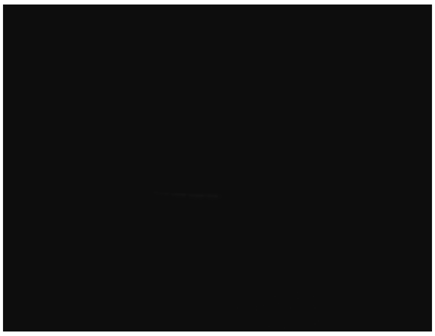

B AMPK

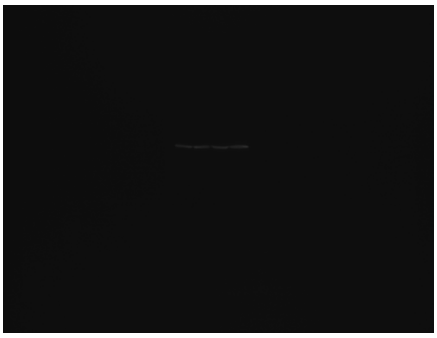

B AMPK GAPDH

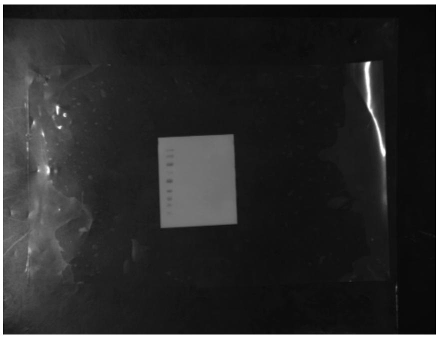

C AMPK marker

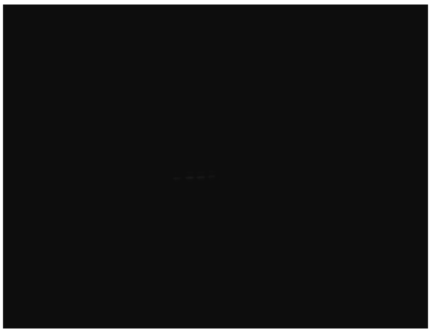

C AMPK

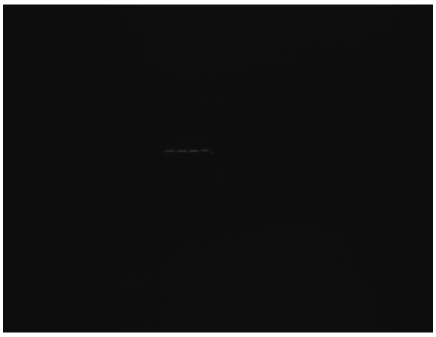

C AMPK GAPDH

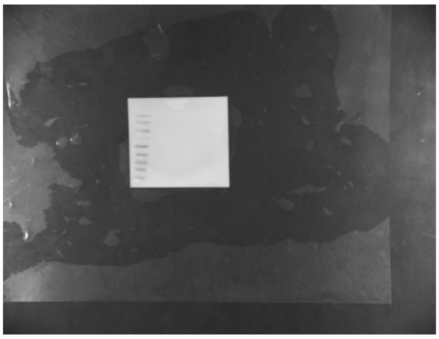

A pAMPK marker

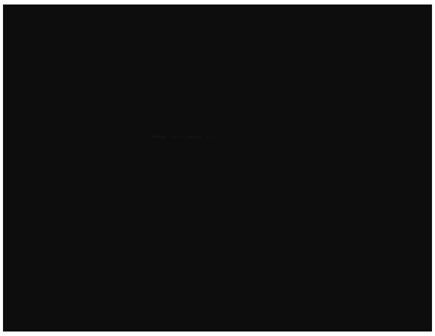

A pAMPK

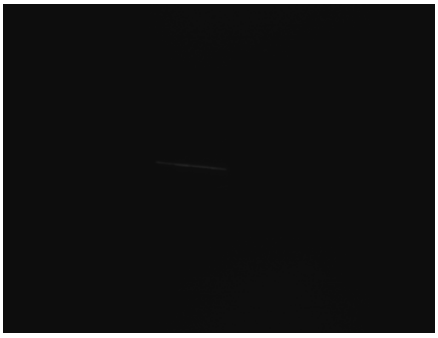

A pAMPK GAPDH

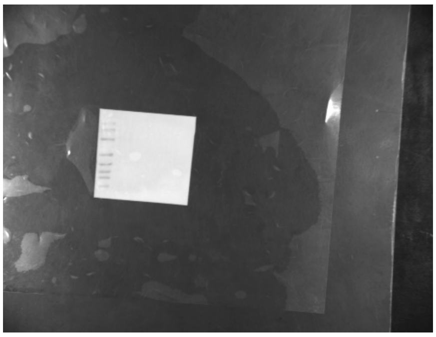

B pAMPK marker

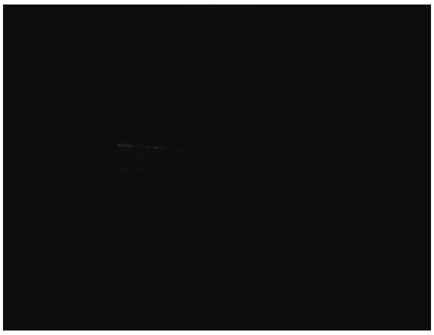

B pAMPK

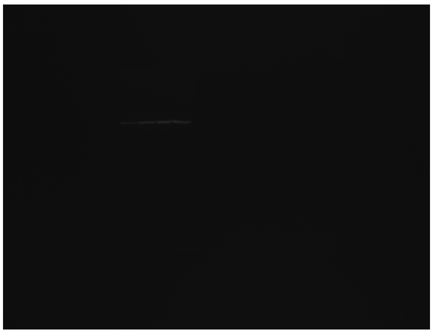

B pAMPK GAPDH

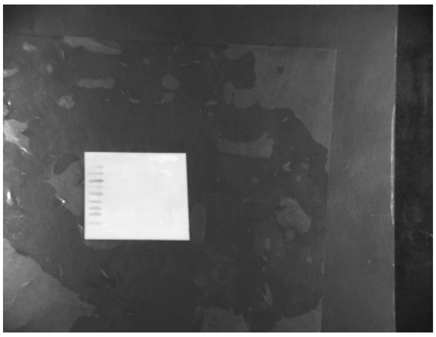

C pAMPK marker

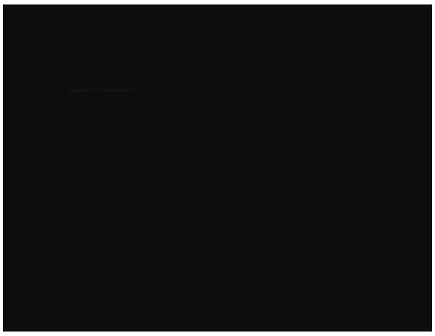

C pAMPK

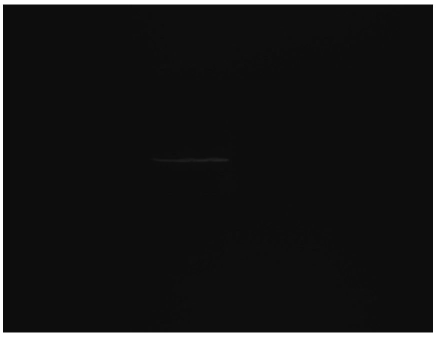

C pAMPK GAPDH

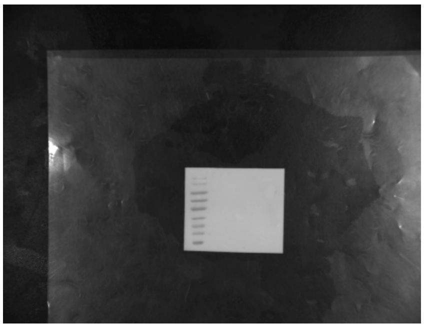
A PGC-1α marker

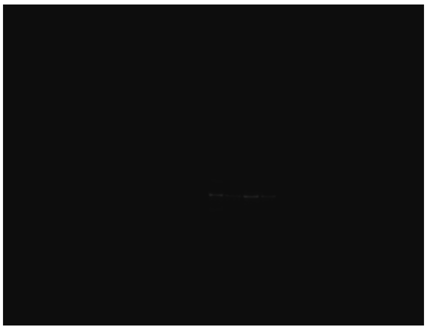
A PGC-1α

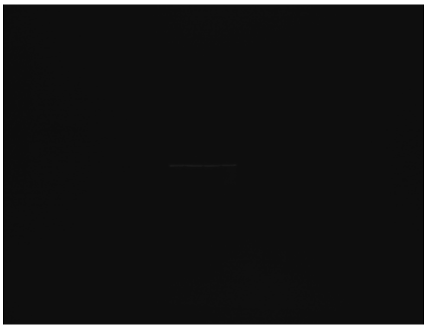
A PGC-1α GAPDH

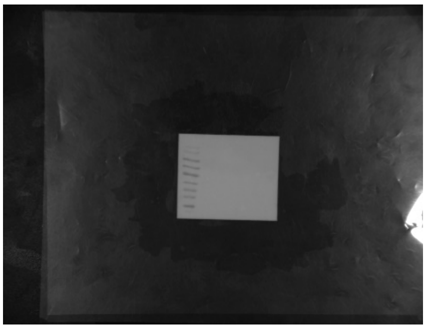
B PGC-1α marker

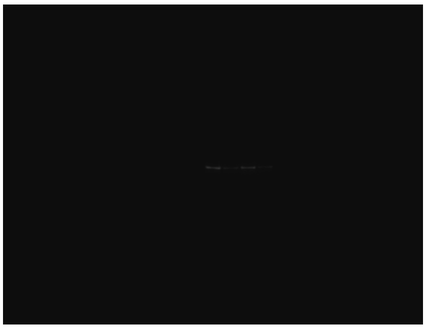
B PGC-1α

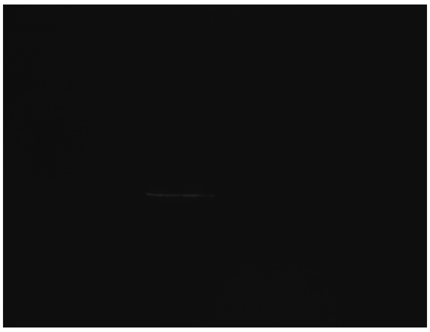
B PGC-1α GAPDH

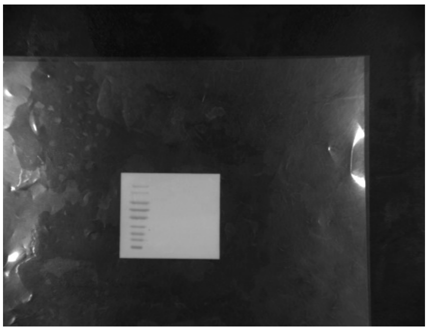
C PGC-1α marker

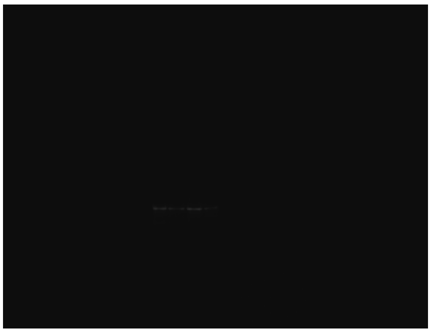
C PGC-1α

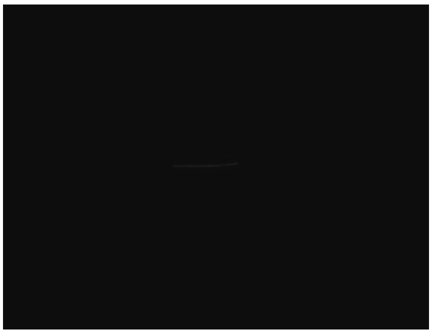
C PGC-1α GAPDH

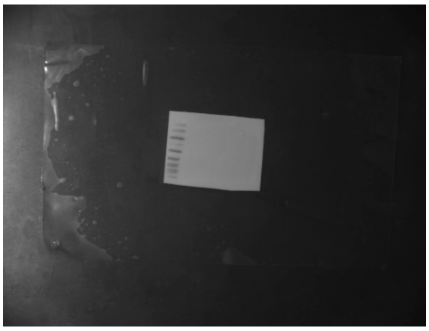
A Sirt1 marker

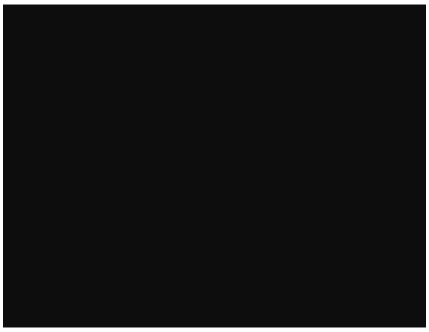
A Sirt1

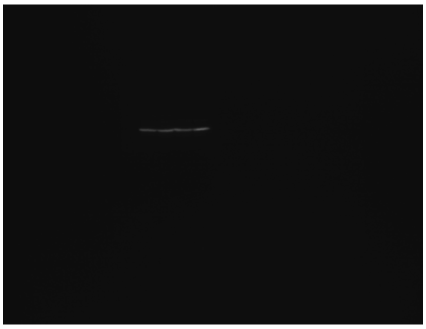
A Sirt1 GAPDH

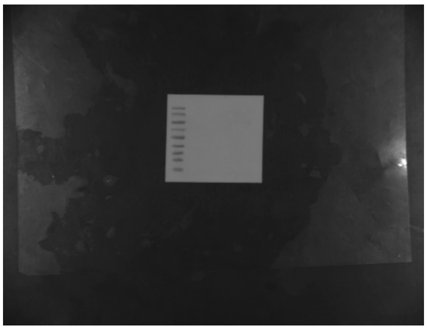
B Sirt1 marker

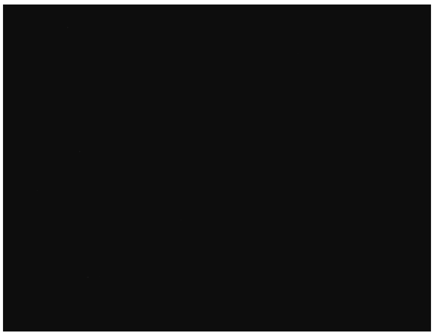
B Sirt1

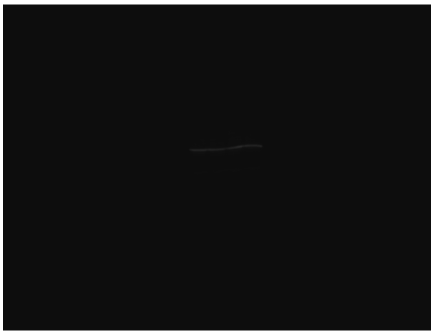
B Sirt1 GAPDH

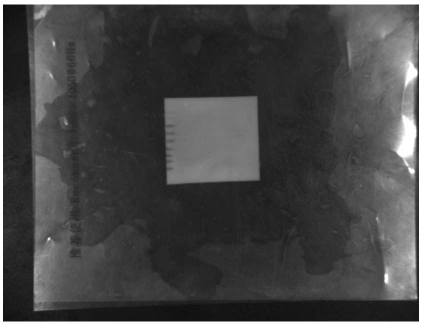

C Sirt1 marker

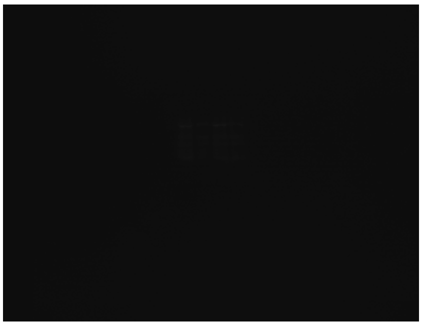

C Sirt1

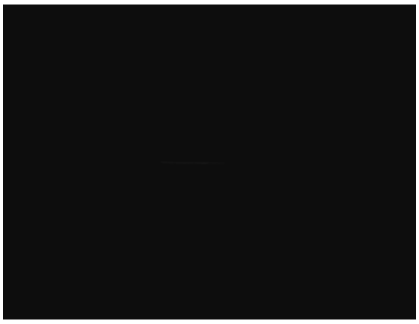

C Sirt1 GAPDH

WB pAMPK/AMPK

Sham	Model	LGSGT	Captopril
0.801264	0.356258	1.188642	0.379288
1.043980	0.303519	0.887955	0.224307
1.203462	0.192476	0.750245	0.552560

WB PGC-1α

Sham	Model	LGSGT	Captopril
0.999684	0.256258	1.262765	0.482752
0.901264	0.314199	1.124749	0.542557
1.136350	0.370437	0.890245	0.644307

WB Sirt1

Sham	Model	LGSGT	Captopril
1.06674	0.139860	0.960784	0.470783
0.80785	0.303045	0.585770	0.270459
0.99409	0.408543	0.770966	0.403774
